# S‐acylation mediates Mungbean yellow mosaic virus AC4 localization to the plasma membrane and in turns gene silencing suppression

**DOI:** 10.1371/journal.ppat.1007207

**Published:** 2018-08-01

**Authors:** Anna Vittoria Carluccio, Maria Isabella Prigigallo, Tabata Rosas-Diaz, Rosa Lozano-Duran, Livia Stavolone

**Affiliations:** 1 Istituto per la Protezione Sostenibile delle Piante, Consiglio Nazionale delle ricerche, Bari, Italia; 2 International Institute of Tropical Agriculture, Ibadan, Nigeria; 3 Shanghai Center for Plant Stress Biology, CAS Center for Excellence in Molecular Plant Sciences, Chinese Academy of Sciences (CAS), Shanghai, China; 4 Chinese Academy of Sciences–John Innes Centre Center of Excellence for Plant and Microbial Science, Shanghai Institutes for Biological Sciences, Chinese Academy of Sciences, Shanghai, China; Agriculture and Agri-Food Canada, CANADA

## Abstract

RNA silencing plays a critical role in plant resistance against viruses. To counteract host defense, plant viruses encode viral suppressors of RNA silencing (VSRs) that interfere with the cellular silencing machinery through various mechanisms not always well understood. We examined the role of *Mungbean yellow mosaic virus* (MYMV) AC4 and showed that it is essential for infectivity but not for virus replication. It acts as a determinant of pathogenicity and counteracts virus induced gene silencing by strongly suppressing the systemic phase of silencing whereas it does not interfere with local production of siRNA. We demonstrate the ability of AC4 to bind native 21–25 nt siRNAs in vitro by electrophoretic mobility shift assay. While most of the known VSRs have cytoplasmic localization, we observed that despite its hydrophilic nature and the absence of trans-membrane domain, MYMV AC4 specifically accumulates to the plasma membrane (PM). We show that AC4 binds to PM via S-palmitoylation, a process of post-translational modification regulating membrane–protein interactions, not known for plant viral protein before. When localized to the PM, AC4 strongly suppresses systemic silencing whereas its delocalization impairs VSR activity of the protein. We also show that AC4 interacts with the receptor-like kinase (RLK) BARELY ANY MERISTEM 1 (BAM1), a positive regulator of the cell-to-cell movement of RNAi. The absolute requirement of PM localization for direct silencing suppression activity of AC4 is novel and intriguing. We discuss a possible model of action: palmitoylated AC4 anchors to the PM by means of palmitate to acquire the optimal conformation to bind siRNAs, hinder their systemic movement and hence suppress the spread of the PTGS signal in the plant.

## Author summary

Plants have developed small RNA (siRNA)-mediated post-transcriptional gene silencing as a defense mechanism against viruses. In response, plant viruses encode viral suppressors of RNA silencing (VSRs) that can interfere with various steps of the silencing pathway. *Mungbean yellow mosaic virus* (MYMV) is a plant bipartite geminivirus responsible for a devastating disease in some areas of tropics and sub-tropics where its natural host, *Vigna mungo*, is a staple food crop. We discovered that the MYMV-encoded AC4 protein is a determinant of pathogenicity, binds native 21–25 nt siRNAs in vitro, and counteracts virus induced gene silencing by strongly suppressing the systemic phase of silencing but not the local production of siRNA. MYMV AC4 undergoes palmitoylation, a post-translational modification never reported before for plant viral proteins that mediates specific localization of the protein to the plasma membrane (PM). Interestingly, palmitoylation and in turns PM localization is indispensable for direct VSR function of AC4. AC4 also binds the PM-located BAM1, a trigger of cell-to-cell spread of RNAi. Taken together our results suggest that AC4 has synergic mechanisms of action, based on the specific PM localization, to prevent spreading of antiviral RNAi silencing in not yet infected cells.

## Introduction

Viruses are obligate intracellular parasites that exploit host machineries to propagate and spread in the host. Their presence and activity deploy diverse plant mechanisms to combat viral infections at both the cellular and the whole-organism levels. Double-stranded (ds)RNA forming during virus replication and self-complementary foldback RNAs from single-stranded viral RNAs or aberrant RNAs can trigger host defence responses via a mechanism of RNA interference (RNAi) that results in inhibition of target RNA expression [1, 2]. The RNase III-type DICER enzymes process these viral RNAs into small-interfering (si)RNAs (21–24 nucleotides) that accumulate in the infected cells and guide the RNA-induced silencing complex (RISC) to degradation of complementary viral RNA sequences [3, 4]. RNA silencing is a non-cell autonomous process thus, silencing signals spread from the site of induction to neighbouring cells and systemically to confer silencing of homologous targets in distant tissues of the host plant [5, 6]. However, the evidence that virus infection often induces symptom and damage in the host highlights the presence of a counter defence strategy that suppresses the host surveillance [7]. Viruses encode one or more proteins that can inhibit initiation (viral RNA recognition and the subsequent degradation), maintenance, or systemic spreading of silencing thus allowing efficient viral replication in single cells and spread of the infection. These virulence factors, called viral suppressors of RNAi (VSRs), share no obvious sequence homology with each other and follow distinct mechanisms of suppression by targeting different points of the RNA silencing pathway, such as viral RNA recognition, dicing, RISC assembly, RNA targeting, and amplification [2, 8]. To overcome the host silencing machinery, several virus species have developed a siRNA sequestration strategy that the different VSR apply in various manners by preventing the assembly of the RISC effector [8]. As siRNA duplexes act as mobile silencing signals moving ahead of the virus to activate antiviral silencing in not yet infected cells, by sequestering and inactivating siRNA VSRs can counter react this defense strategy and allow spreading of the viral infection in the plant [9].

Members of the family *Geminiviridae* are small, circular, single-stranded DNA viruses composed of one or two genomic segments of 2500–3100 nucleotides which are encapsidated within small twinned icosahedral particle that replicate in the nucleus of an infected cell via double-stranded intermediates that also serve as templates for bidirectional transcription [10].

Geminivirus host different suppressor proteins encoded by open reading frame (ORF) AC2, V2, ORF **β** C1, ORFs AC4 and AC5 [11–15]. The transcriptional activator protein (TrAP) encoded by the ORF AC2 of *African cassava mosaic virus* (ACMV), *Tomato golden mosaic virus* and *Mungbean yellow mosaic virus* (MYMV), the C2 of Tomato leaf curl virus and the **β** C1 of the *Tomato yellow leaf curl China virus* (TYLCCNV) share sequence nonspecific DNA binding activity and localization in the nucleus where they act by a mechanism depending on interaction with DNA and transcriptional activation or with key components of the RNA silencing pathway. On the other side, the V2 protein of *Tomato yellow leaf curl virus* (TYLCV)-Is has specific cytoplasmic localization and exerts its VSR activity by targeting a step after siRNA production thus representing a different example of VSR in geminivirus [16]. The MYMV AC5, a protein encoded by some begomoviruses, suppresses post-transcriptional gene silencing (PTGS) and can reverse methylation-mediated TGS [14].

The AC4/C4 gene lies entirely within the Rep coding region, but in a different reading frame, and is one of the least conserved among members of the *Geminiviridae* family. Its function is very controversial: mutagenesis and/or transgenic expression of some AC4/C4 genes results in no phenotype or phenotypes consistent with movement protein or symptom determinant activity [17]. This puzzling information has been enriched with the discovery of a role of AC4/C4 in the suppression of RNA silencing in different strains of ACMV [13, 18], in MYMV [19], in the monopartite TYLCV and in *Bhendi yellow vein mosaic virus* [20, 21]. These proteins block cytoplasmic RNA silencing by a mechanism that involves binding of single-stranded siRNA and miRNA and possibly facilitates their degradation. This suggests that the severe developmental defects observed upon transgenic expression of some AC4/C4 might be due to suppression of overlapping steps in the siRNA and miRNA pathways [22, 23].

Interestingly, AC2s and AC4s of cassava viruses behave differently in regulating silencing suppression exerting strong or weak activity depending on the viral strain, and apparently compensating each-other function [13]. Consequently, mixed-strain infections can be responsible of unusually severe cassava mosaic disease in the field [24].

S-acylation or palmitoylation, is a reversible posttranslational modification of a protein covalently attaching through a cysteine residue(s) to long chain fatty acid, usually the 16-carbon palmitate via a thioester bond. This modification increases protein membrane affinity and provides an important mechanism for regulating cellular functions including subcellular localization, stability, trafficking, stress response, disease resistance, hormone signaling, cell polarisation, cell expansion and cytoskeletal organization [25, 26].

Unique among lipid modifications of proteins, this attachment is reversible, thus offering dynamic control over the cellular processes and protein function in response to stimuli. Our understanding of S-acylation function in plants is quite limited compared with other organisms and mainly comes from targeted studies on the functional characterization of individual proteins that happen to be S-acylated [27].

Several examples both in plant and animal systems describe palmitoylation as a modification used by proteins to switch subcellular localization between nucleus and plasma membrane (PM) and to accomplish their tasks. For example, specific functions regulated by transcription factor (TF) in the nucleus are triggered or hindered by palmitoylation-mediated protein localization to the nucleus or to the PM, respectively [28, 29].

In plants, differential subcellular localization of TF induced upon palmitoylation, are associated to plant response to abiotic stresses such as salt and drought increase [28, 30]. A large number of S-acylated proteins are also involved in plant–microbe interactions. Among them, a proteomic approach identified proteins involved in pathogen perception and response, mitogen-activated protein kinases (MAPKs), leucine-rich repeat receptor-like kinases (LRR-RLKs) and RLK superfamily members, ATPases, integral membrane transporters, soluble N-ethylmaleimide-sensitive factor-activating protein receptors (SNAREs) and heterotrimeric G-proteins [31]. PM is a critical subcellular compartment for the actors of a pathosystem. In fact, plants use the covalent addition of fatty acids to target an array of sensor/receptor proteins to the PM and detect invading pathogens whereas pathogens (except for viruses that do not penetrate plant cells actively) secrete effector proteins into the plant cell, particularly the internal face of the host PM, to threaten this surveillance and induce plant susceptibility to infection [32]. Animal viral proteins such as glycoproteins from *Vesicular stomatitis virus* [33], the *Influenza virus* hemagglutinin as well as the transmembrane Matrix-2 (M2) [34, 35] can also undergo S-acylation that is essential for virus replication or infection. However, palmitoylation of plant viral proteins has not been reported so far. Interestingly, C4 protein from Tomato yellow leaf curl virus (TYLCV) targets, through a yet unknown mechanism, PM and plasmodesmata (PD) where inhibits the intercellular spread of RNAi by interacting with receptor-like kinase (RLK) BARELY ANY MERISTEM 1 (BAM1) [36].

In this study, we examined the role of MYMV AC4 in viral infectivity. We revealed that AC4 undergoes post-translational palmitoylation that mediates protein targeting to the PM. When localized to the PM, AC4 strongly suppresses systemic silencing whereas delocalization from such subcellular compartment impairs VSR activity.

## Results

### AC4 is essential for systemic infection of MYMV and symptom development

As a first step to gain more insights into the function of MYMV AC4, we determined whether AC4 is essential for successful MYMV infection. To this aim, we modified the three in frame start codons of the ORF AC4 within the infectious clone pGA1.3A [37] to obtain a MYMV-ΔAC4 DNA A mutant. Mutations were designed to be silent in the overlapping AC1 ORF and did not produce any change in the amino acid sequence of AC1. *Vigna mungo* plants were biolistically inoculated with recombinant and wild type (wt) MYMV DNA A, each together with the infectious MYMV DNA B clone pGA1.3B [37], and monitored for symptom appearance for two months.

**Fig 1:**
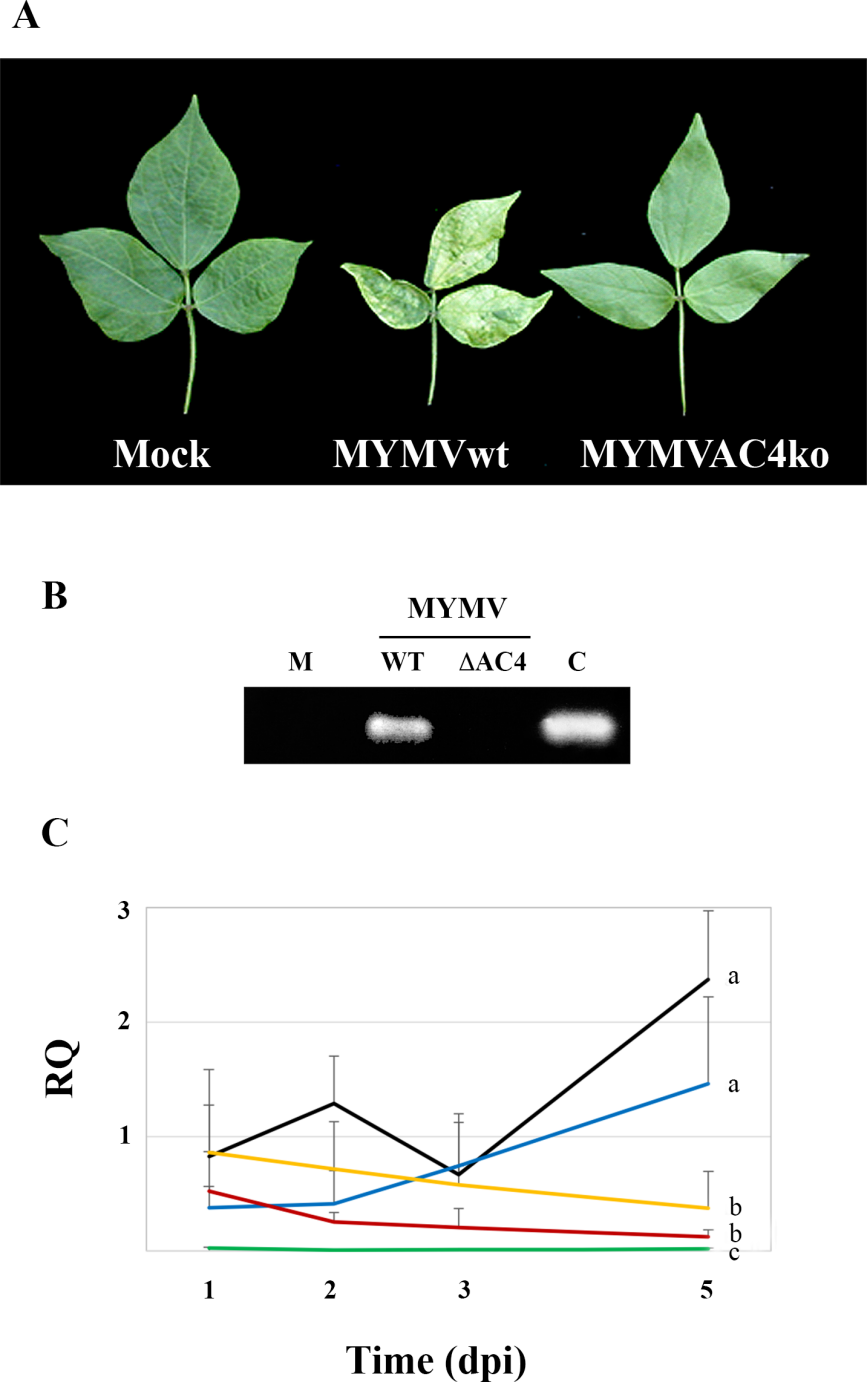
Development of MYMV-△AC4 infection in *V*. *mungo* plants. (A) Systemic symptoms on plants inoculated with MWMV WT and MWMVΔAC4 mutant observed 4 weeks after virus inoculation. (B) PCR amplification of the AC4 gene from total DNA extracts of systemic leaves. M: mock inoculated plants; C, control: plants inoculated with MYMV DNA A alone. (C) Quantification of MYMV accumulation by qPCR of the AC2 gene, from 1 through five days after *V*. *mungo* inoculation with MYMV wt (black line), its mutant variants MYMVΔAC4 (blue line), MYMVΔBC1 (yellow line) and MYMVΔAC1 (red line), and the negative control pCKEGFPAC2 (green line). RQ: relative quantity; Time: day post inoculation. Data are linked by tendency line, each value is the mean of three biological replicates and two independent experiments, and RQ are normalized to the expression levels of endogenous *V*. *mungo* actin mRNA. Bars are 95% confidence intervals. Treatments with different letters are significantly different according to interaction contrast tests.

Typical yellow mosaic and leaf curling symptoms in the trifoliate leaves became evident 18 (+/- 3) days post inoculation (dpi) on plants infected with wt MYMV whereas no symptom were observed on plants inoculated with a MYMV-ΔAC4 for the entire time of observation (Fig 1A). PCR analysis of total DNA extract confirmed the absence of viral DNA in systemic leaves (Fig 1B) and highlighted the requirement of AC4 for systemic spread of MYMV in the host.

### MYMV accumulation in infected *V*. *mungo* leaves

The absence of systemic symptoms in plants inoculated with MYMVΔAC4 might be either consequence of a local event (such as virus inability to replicate/accumulate in the initially inoculated cells or to move out of them) or reflect long distance movement incompetence and, similar to homologue geminivirus VSR, MYMV AC4 could suppress very early host antiviral defence [13].

To investigate in more detail the role of AC4 in the early stage of infection, we conducted quantitative real-time (qPCR) experiments to analyse and monitor the accumulation of MYMV in *V*. *mungo* biolistically-inoculated leaves during the initial five days of infection. To this aim, we constructed two control virus mutants: MYMVΔAC1 and MYMVΔBC1 expressing null replicase and movement protein functions, respectively. To evaluate the genome replication and accumulation of MYMVΔAC4, MYMVΔAC1 and MYMVΔBC1 virus mutants relatively to MYMV WT, we compared the quantification of the MYMV AC2 gene, unrelated to the mutated genes, at four time points (1-2-3-5 dpi) and used contrast statistical analysis to compare the value recorded at 5 dpi with those obtained at the previous time points for each couple of virus constructs inoculated.

Statistically equivalent trends of viral DNA accumulation were observed in plants inoculated with MYMV WT and MYMVΔAC4, both of which reached the highest and statistically most distinct value at 5 dpi (Fig 1C).

Due to the lack of viral replicase, the concentration of MYMVΔAC1 dropped within the first two days and continued to decrease slowly but constantly accordingly to the continuous degradation of the input DNA (Fig 1C, red line).

Accumulation of the MYMVΔBC1 mutant, deprived of the movement protein function, also followed a negative trend statistically not different from MYMVΔAC1 (Fig 1C). However, differently from MYMVΔAC1, the concentration of MYMVΔBC1 decreased more slowly than MYMVΔAC1, probably reflecting the occurrence of MYMVΔBC1 replication in single cells, and remained statistically not different from MYMVΔAC4 until 3dpi (S1 Fig). From this time point, the inability to exit infected cells probably triggered MYMVΔBC1 DNA degradation or infected-cell death.

Taken together, these results, obtained from three independent replications of the experiment, indicate that, in the absence of AC4, MYMV can replicate and move from cell to cell, and rule out the absolute requirement of AC4 for virus replication and cell-to-cell movement. However, the lower efficiency of MYMVΔAC4 compared to wt (S1 Fig), and the relatively low, albeit statistically significant difference with the movement-impaired MYMVΔBC1 mutant, suggest that a possible indirect contribution to the mechanism of virus transport in plant cannot be excluded.

### MYMV AC4 is a determinant of PVX symptoms in *N*. *benthamiana*

Many VSRs are pathogenic determinants of their virus host. They can interfere, independently or synergistically with other VSRs, to enhance the severity of symptoms caused by related or unrelated viruses [38, 39]. As the knock out of AC4 in the viral genome hinders systemic plant infection, to investigate the involvement of this VSR in viral pathogenicity, we tested the effect of MYMV AC4 expression on the symptom onset induced by heterologous *Potato virus X* (PVX) [40] infection in *Nicotiana benthamiana*.

**Fig 2:**
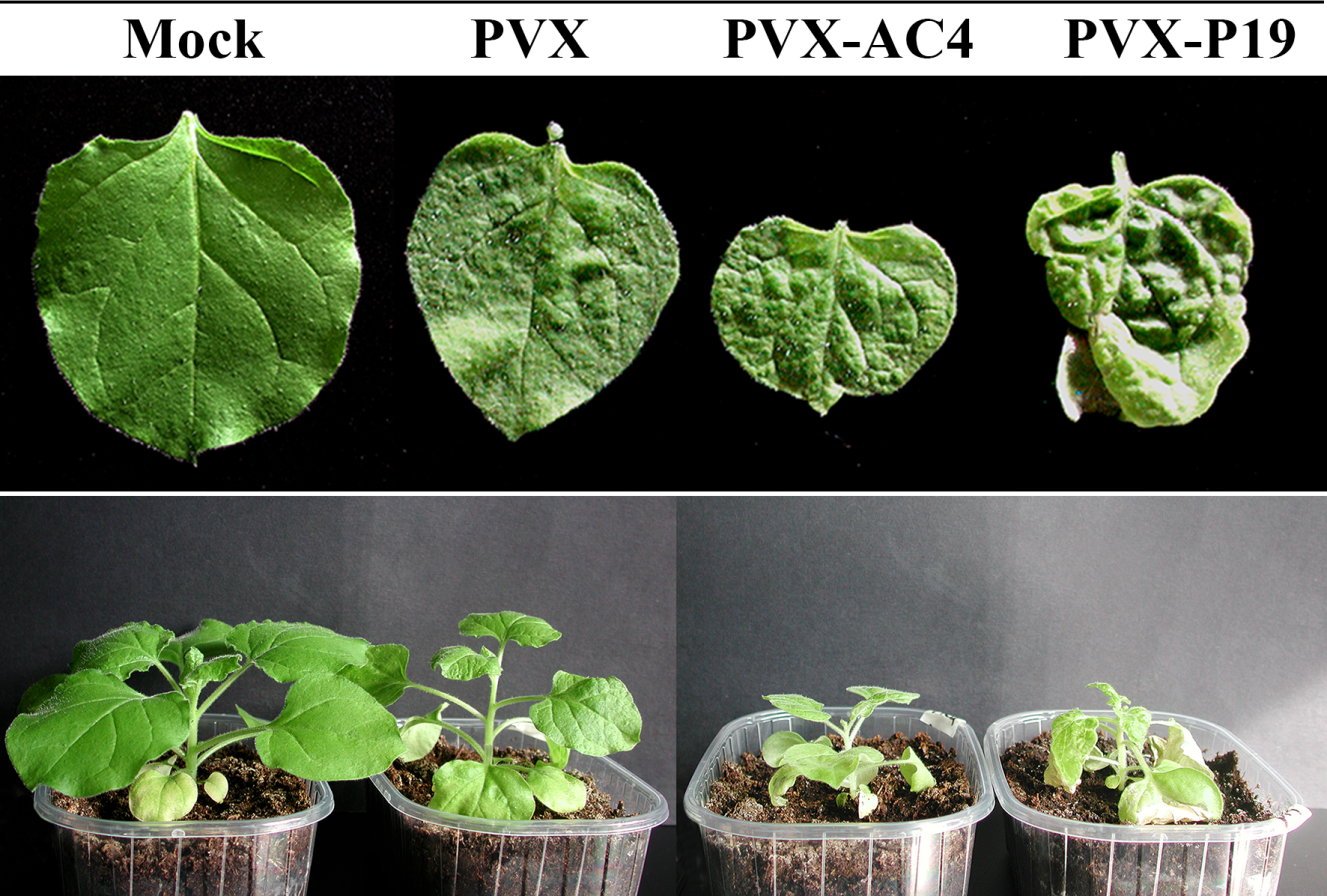
*N*. *benthamiana* plants agroinoculated with MYMV-encoded AC4 from the PVX vector. Plants agroinfiltrated with: Mock, agrobacterium (no PVX); PVX, empty virus vector; PVX-AC4, PVX-vector containing AC4 ORF; PVX-P19, PVX-vector containing P19 ORF. Images correspond to 10dpi. P19 derives from *Carnation Italian ringspot virus* and is used as positive control.

All inoculated plants developed systemic leaf puckering and those expressing VSRs showed additional severe stunting (Fig 2). PVX-induced symptoms were significantly worsened by the simultaneous expression of AC4; however, the necrotic phenotype typically induced by *Carnation Italian ringspot virus* P19, which we used as positive control [41, 42], was not observed in plants agroinfiltrated with PVX-AC4 (Fig 2). This result provides support for the notion that MYMV AC4 is a determinant of viral pathogenicity and suggests that, as for other VSR, the enhancement of PVX-induced symptoms might be related to the capacity of AC4 to interfere with components of the endogenous RNAi pathway.

### AC4 is a suppressor of VIGS

Aiming at understanding the molecular basis of the RNAi suppression activity of AC4, we used GFP-transgenic *N*. *benthamiana* plants (line 16c) overexpressing GFP constitutively [39]. These plants show green fluorescence under UV light. Upon transient expression of GFP, inducing silencing of the transgenically expressed GFP, they display only chlorophyll autofluorescence and appear red under UV light. In the presence of a suppressor of RNA silencing GFP silencing is blocked and plants continue to exhibit green fluorescence.

**Fig 3:**
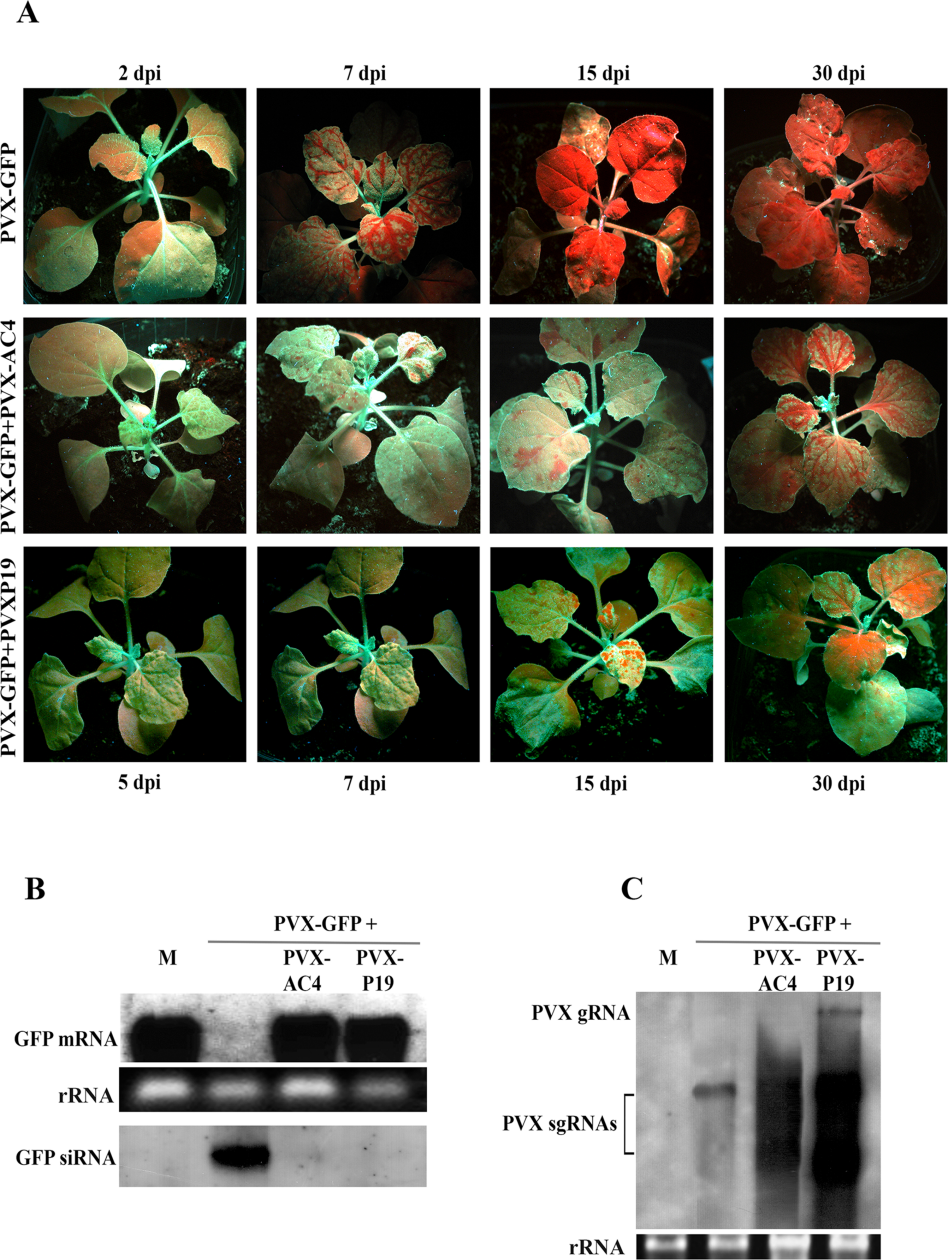
Effect of MYMV AC4 on PVX-induced GFP silencing in *N*. *benthamiana* 16c. (A) Plants agroinfiltrated with PVX-GFP and PVX-GFP+PVX-AC4. Constructs and time points are shown at the left side and the top/bottom of the images, respectively. Northern blot detection of (B) GFP mRNA, siRNA and (C) PVX coat protein mRNA extracted from *N*. *benthamiana* 16c leaf tissues agroinfiltrated with PVX-GFP, PVX-GFP+PVX-AC4, PVX-GFP+PVXP19 at 15 dpi. Constructs are shown at the top of the images. M, mock-agroinfiltrated plants; gRNA, genomic RNA; sgRNA, sub-genomic RNA; rRNA represents ethidium bromide-stained ribosomal RNA shown as loading control.

To induce silencing, we used a PVX-GFP plasmid [40] to agroinfiltrate *N*. *benthamiana* 16c plants and monitored the progress of silencing on the upper leaves for 30 days. Upon infiltration of PVX-GFP, ectopic GFP expression was observed in the inoculated leaves and in the veins of new leaves from 2 dpi under UV light (Fig 3A). The intensity of the green fluorescence signal increased until 5 dpi but was followed by a rapid replacement of the green fluorescence with chlorophyll red autofluorescence, due to GFP silencing, at 6–7 dpi (Fig 3A). By 12 dpi these plants appeared completely red fluorescent (Fig 3A).

Plants inoculated with equal amounts of PVX-GFP, PVX-AC4 (PVX-GFP+PVX-AC4) and PVX-P19 (PVX-GFP+PVX-P19) showed green fluorescence on newly emerging leaves starting from 5 dpi, simultaneously to the onset of silencing in PVX-GFP agroinfiltrated plants (Fig 3A).

At 15 dpi, when PVX-GFP plants were completely red fluorescent, plants infiltrated with PVX-GFP+PVX-AC4, similar to the control and PVX-P19 (PVX-GFP+PVX-P19) [42], were still green fluorescent, and most of the leaves continued to show silencing suppression at 30 dpi, very strongly in the youngest emerging leaves (Fig 3A).

RNA gel blot analysis of GFP mRNA and 21–25 nt RNA confirmed these observations. 15 dpi, high levels of GFP mRNA were found in young leaves of plants inoculated with both PVX-GFP+PVX-AC4 and with PVX-GFP+PVX-P19 (used as positive control) whereas GFP mRNA was undetectable in plants inoculated with PVX-GFP (Figs 3B and 6C). Conversely, GFP siRNA where detected only in plants inoculated with PVX-GFP alone (Fig 3B).

Collectively, these results demonstrate that co-delivery of GFP and MYMV AC4 onto GFP-expressing *N*. *benthamiana* strongly suppresses the onset of VIGS compared with the progress of gene silencing obtained with PVX-GFP alone.

A Northern blot analysis was also conducted on the same samples with a PVX coat protein (CP) probe. Consistent with the extent of silencing suppression, very high levels of PVX chimera were observed in plants inoculated with PVX-GFP+PVX-AC4 and with the PVX-GFP+PVX-P19 control (Fig 3C). The fact that the PVX CP probe detected much lower levels of viral RNA in plants inoculated with PVX-GFP alone (Fig 3C) suggests that the spread of GFP silencing observed in young leaves of plants inoculated also with PVX-GFP+PVX-AC4 was due more to the spread of the silencing signal than to a de novo silencing by PVX-GFP.

These results demonstrate that MYMV AC4 can suppress silencing-related defence responses in transgenic *N*. *benthamiana* plants. Nevertheless, the loss of virus accumulation observed in systemic leaves of *V*. *mungo* infected with MYMV-ΔAC4 (Fig 1B) suggests that this may be the result of systemic VIGS also in the virus natural host.

### AC4 suppresses systemic but not local PTGS

**Fig 4:**
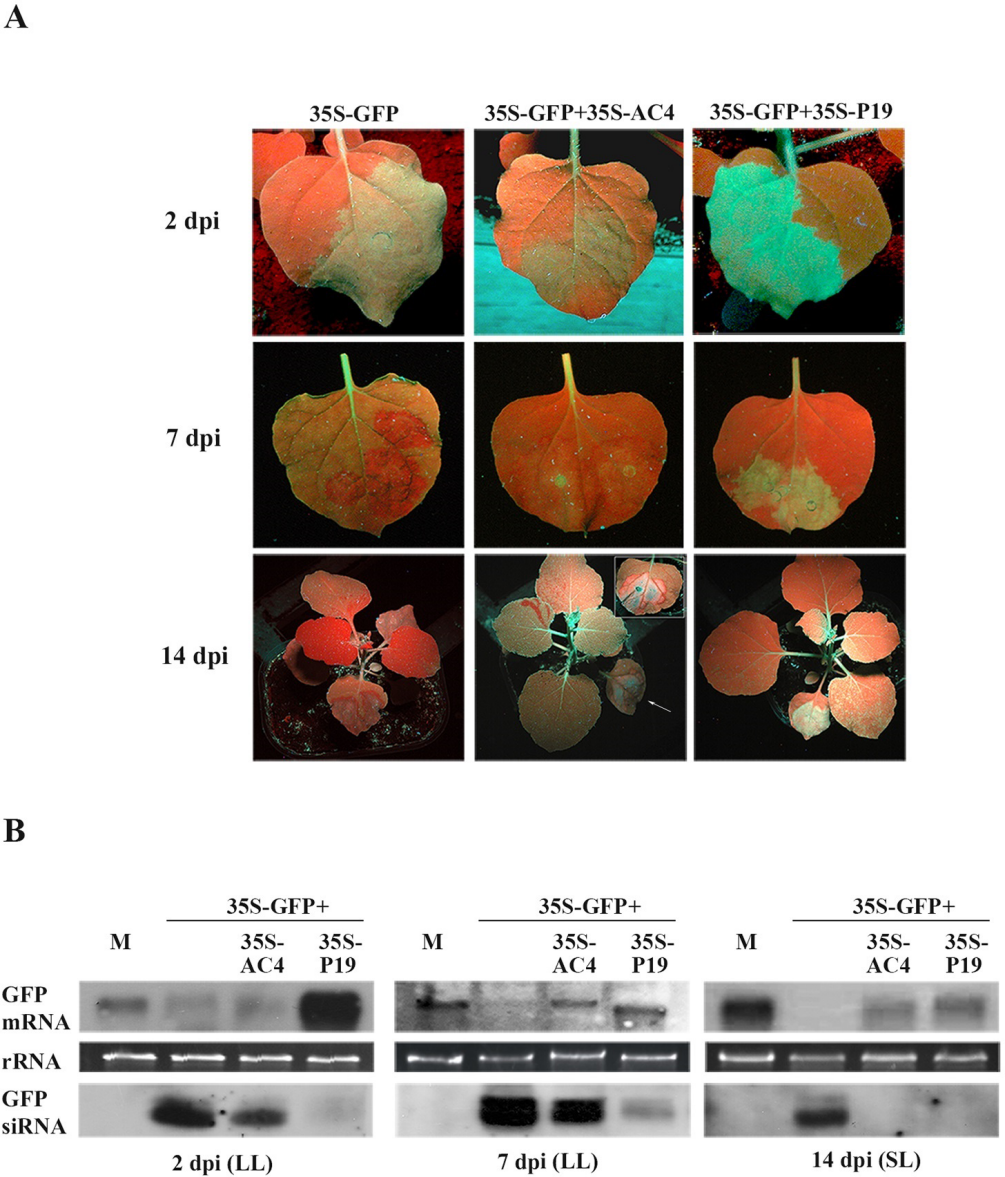
MYMV AC4 suppression of transgene-induced PTGS. (A) Accumulation of green fluorescence in leaves (top and middle panels) and plants (bottom panel) of *N*. *benthamiana* 16c infiltrated with 35S-GFP alone or in combination with 35S-AC4 or 35S-P19. Constructs and time points are shown at the top and left side of the images, respectively. (B) GFP mRNA and siRNA extracted from infiltrated (LL) and systemic (SL) leaves of the plants illustrated above. Constructs and time points are shown at the top and bottom of the images, respectively. M, mock-agroinfiltrated plants. rRNA represents ethidium bromide-stained ribosomal RNA shown as loading control.

The VSR activity of AC4 was further investigated in PTGS experiments. To this aim, AC4 and P19 were cloned under the control of the 35S promoter and each co-infiltrated to line 16c plants, together with the silencing inducer (full-length GFP) under the control of the same promoter. Two dpi, infiltrated leaf patches appeared green fluorescent under UV light and, consistently with higher accumulation of the GFP transcript, those co-infiltrated with the P19 VSR control appeared brighter than the others (Fig 4A and 4B). By 7 dpi, when red fluorescence had completely replaced green fluorescence in 35S-GFP infiltrated patches, GFP mRNA was still present in AC4 and P19 co-agroinfiltrated plants but almost undetectable in plants infiltrated with 35S-GFP alone (Fig 4A and 4B).

Interestingly, GFP siRNAs started to accumulate in 35S-AC4 co-infiltrated patches at 2 dpi, and at 7 dpi they were in a concentration similar to 35S-GFP but much higher than the 35S-P19 control (Fig 4B). Remarkably, despite a red fluorescent front developed around the infiltrated area, systemic GFP silencing as well as accumulation of 21–25 nt RNA were not observed in the upper leaves of plants agro-infiltrated with 35S-GFP+35S-AC4 (Fig 4A and 4B).

The evidence that siRNAs accumulate in 35S-GFP+35S-AC4 patches at a concentration similar to 35S-GFP (Fig 4B) reveals that MYMV AC4 does not interfere with production of transgene-induced gene silencing whereas the absence of siRNAs in the upper leaves indicates a possible involvement in long-distance spreading of the silencing signal.

### MYMV AC4 has a double localization in the cell

**Fig 5:**
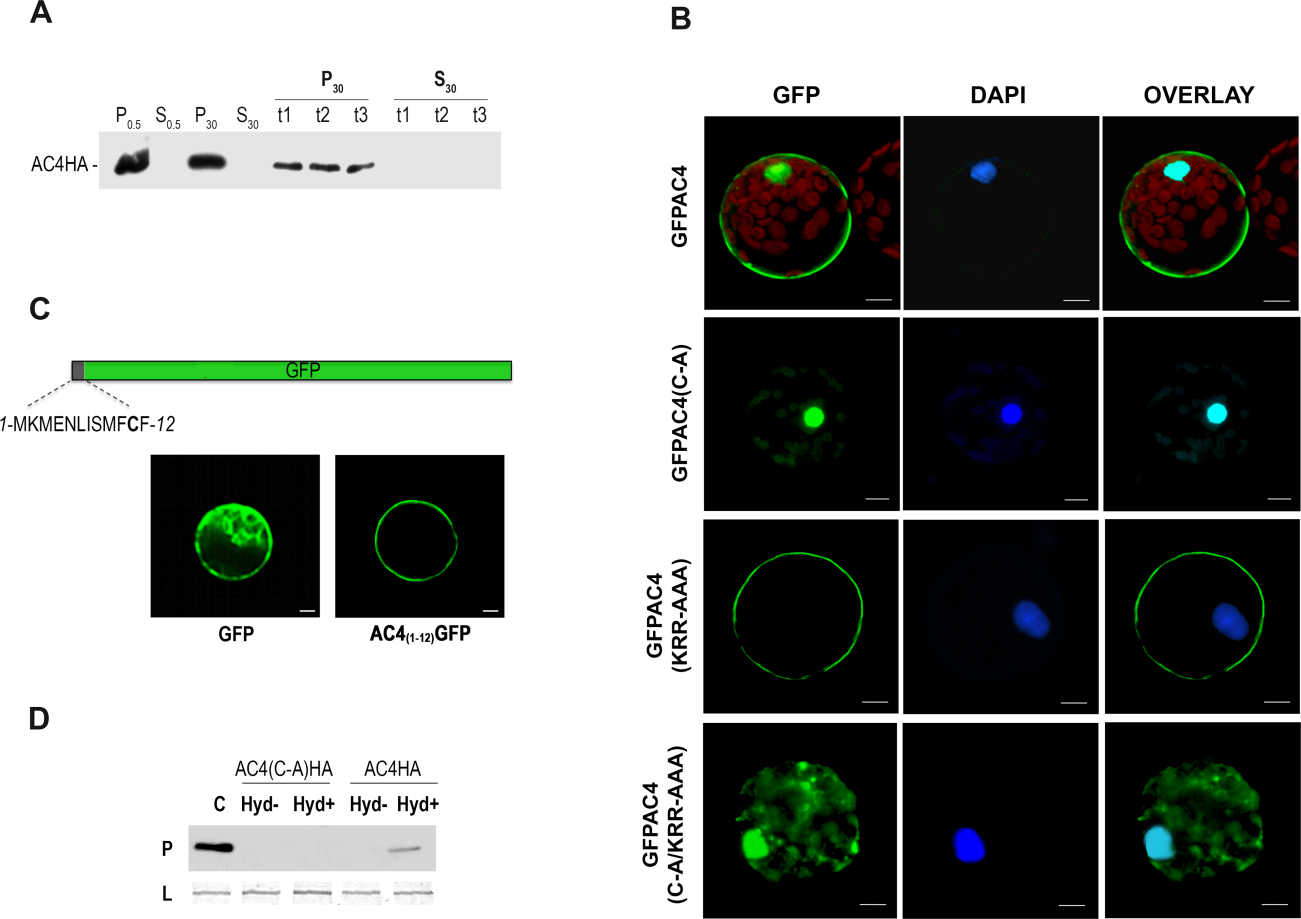
Subcellular localization of AC4 and its mutant variants. (A) Western blot analysis of protein extracts from protoplasts transfected with pCKAC4HA and fractionated in a 500 X g (0.5) and a 30,000 X g (30) pellet (P) and supernatant (S). Lanes 1–4 represent untreated extracts. t1, t2 and t3 represent pellet from high-speed centrifugation of the same protein extracts further treated with Na_2_CO_3_, Urea, and KCl, respectively. AC4 is revealed with anti-HA antibody. (B) Transient expression of GFP-fused wild type AC4 (GFPAC4) and its mutant variants (C-A, KRR-AAA, and C-A/KRR-AAA) in *N*. *benthamiana* protoplast and DAPI-stained nuclear DNA. If GFP-AC4 and mutants are localized to the nucleus (dark blue), the latter appears light blue in the merge images (Overlay). Bars = 20 **μ**m. (C) Transient expression of GFP-fused with N-terminal 12 amino acids of AC4 in *N*. *benthamiana* protoplast. The AC4_(1–12)_GFP construct is depicted by boxes. GFP, represents expression of free wt GFP. (D) Western immunoblotting for detection of AC4HA palmitoylation by biotin switch assay. Protein extracts from *N*. *benthamiana* protoplasts expressing AC4HA and AC4(C-A)HA were treated with (Hyd+) or without (Hyd-) hydroxylamine, the thioester cleavage reagent required for detection of palmitoylated proteins, and subjected to the biotin switch assay. C: AC4HA from untreated protoplast extract; P: Palmitoylation, shows the levels of AC4HA and AC4(C-A)HA recovered from the neutravidin beads, and therefore originally S-acylated; L: loading controls, shows that equal amounts of AC4HA and AC4(C-A)HA were loaded onto neutravidin beads. Proteins are revealed with anti-HA antibody.

To identify the major site(s) of subcellular localization of AC4, we fused the recombinant AC4 with an influenza virus hemagglutinin epitope (HA) tag, and used it for protoplast transfection. Protoplasts transfected with pCKAC4HA were lysed at 24 h post transfection (hpt) and the lysate was submitted to differential centrifugations: low speed centrifugation (500 g) to collect nuclei and residual intact cells, and high speed centrifugation (30000 g) to separate the soluble membrane fraction from the crude part. Equivalent amounts of each fraction were analysed by immunoblotting with an anti-HA antibody. AC4 was detected in the pellets from both low- and high-speed centrifugations (Fig 5A) but was absent in the supernatants. To further investigate the association of AC4 with the membrane fraction, we treated the pellet obtained from high-speed centrifugation with Na_2_CO_3_, urea or KCl that remove proteins weakly bound to membranes. After a second high-speed centrifugation, a band corresponding to AC4 was detected in every pellet regardless of the different treatment applied (Fig 5A). The evidence that none of the treatments could dislodge AC4 from the membrane fraction indicates a very strong interaction of the protein with cellular membranes whereas the presence in the low-speed pellet suggests that AC4 could also be present in the cytosol and possibly in the nucleus.

The subcellular localization of AC4 was further investigated by expressing the protein in fusion with GFP in *N*. *benthamiana* protoplasts. The fluorescence signal was monitored at different time points between 4 and 48 hpt. Between 4 and 6 hpt, a fluorescence signal localized to the PM was visible in most of the transfected protoplasts whereas only in few of them GFP-AC4 was also visible in the nucleus. Starting from 8 hpt the majority of fluorescent protoplasts showed a double localization to the PM and the nucleus (Fig 5B) that did not change in the course of the experiment. GFPAC4 was also expressed in *V*. *mungo* leaf mesophyll by means of biolistic particle delivery. Consistently with observation in protoplasts, GFPAC4 localizes in the nucleus and at the cell periphery of single mesophyll cells (S2 Fig), and accumulates at PD (S3 Fig).

*In silico* analysis of the physical-chemical properties of AC4, performed by using the web-interface SeqWeb of GCG Wisconsin Package (version 2) [43], predicted that AC4 is an hydrophilic protein except for a region comprised between aminoacids 6 to 12 (S4 Fig). The core of this region is characterized by two hydrophobic phenylalanines flanking a polar cysteine (position 11), which, the CSS-*Palm* 4.0 software [44] predicted might be palmitoylated (S4 Fig). The AC4 sequence following this hydrophobic part is expected to have a high surface probability (S2 Fig). The pick of this region is occupied by the KRR amino acid sequence that was predicted to be a potential nuclear localization signal (NLS) by the NucPred software [45].

To gain more insight into the involvement of the two in silico-identified domains in the subcellular localization of AC4, we replaced the amino acid C_11_ by an alanine to produce the GFPAC4(C-A) mutant. The amino acids K_19_, R_20_ and R_21_ were also replaced together by alanines to obtain the mutant GFPAC4(KRR-AAA). These single mutants and a double mutant comprising both mutations GFPAC4(C-A/KRR-AAA), were transiently expressed in *N*. *benthamiana* protoplasts and fluorescence signal was observed at 24 hpt.

Upon alanine-substitution of C_11_, GFPAC4(C-A) accumulated only in the nucleus and didn’t show PM localization at any time (Fig 5B). On the other side, mutation of the hypothetical NLS delocalized AC4(KRR-AAA) from the nucleus and the protein accumulated only at PM (Fig 5B). Mutation of both domains resulted in cytoplasmic diffusion of AC4(C-A/KRR-AAA) with subcellular localization indistinguishable from free GFP (Fig 5B). These results, further supported by similar results obtained upon expression in single mesophyll cells of bombarded *V*. *mungo* leaves (S2 Fig), indicate that the *in-silico* predictions were correct and that the two predicted domains are indeed responsible for the subcellular localization AC4.

### MYMV AC4 is post-translationally palmitoylated

AC4 mutagenesis indicates that C_11_ is a critical amino acid for protein targeting to the PM and strongly supports the *in silico* prediction of post-translational palmitoylation of the protein. S-acylation (palmitoylation) is the reversible post-translational addition of a saturated fatty acids (palmitate or stearate) through thioester linkages to cysteine residues of proteins [46]. While no specific consensus domain exist for palmitoylation, the cysteine involved in the thioester bond should be localized inside the protein in a favourable context to allow insertion of the fatty acid and docking to the PM [47]. To confirm that C_11_ in AC4 can direct protein localization to the PM, we inserted the AC4 DNA sequence encoding aminoacids 1 through 12 upstream of *egfp* (Fig 5C). The corresponding fusion protein, AC4_(1–12)_GFP was transiently expressed in *N*. *benthamiana* protoplasts and observed at 24 hpt by video confocal microscopy. The addition of the N-terminal 12 amino acids of AC4 displaces GFP from te cytosol and AC4_(1–12)_GFP is relocated to the PM (Fig 5C).

To get a definitive proof of AC4 palmitoylation, we performed a biotin-switch assay, a biochemical test using hydroxylamine for specific cleavage of thioester bonds. Therefore, this assay allows only palmitoylated proteins to be cleaved and biotinylated in a cellular lysate and, upon biotin affinity purification, their detection by immunoblotting. To this aim, we transfected protoplasts with the pCKAC4HA or pCKAC4(C-A)HA mutant plasmids expressing recombinant proteins in fusion with the HA tag. The protoplast lysate was subjected to the biotin-switch assay and the presence of AC4-HA in the precipitate, detected by western blotting using an anti-HA antibody (Fig 5D) indicates that AC4 is S-acylated in planta. On the other hand, the evidence that AC4(C-A)HA was not recovered from the neutravidin beads (Fig 5D) reveals that the mutant protein was not originally S-acylated and that C11 is essential for AC4 palmitoylation.

### PM localization of AC4 is essential for VIGS suppression

Even though the site of accumulation of a protein does not necessarily correspond to its site of biological action, the evidence that AC4 is post-translationally modified to specifically target the cellular plasma membrane suggests that a protein function could be connected to palmitoylation. As AC4 acts as a VSR, we investigated the relation of silencing suppression function with PM localization. To this aim, we agroinfiltrated the PVX-GFP vector in combination with PVX-AC4, PVX-AC4(C-A), PVX-AC4(KRR-AAA) or PVX- AC4(C-A/KRR-AAA) in *N*. *benthamiana* 16c plants.

**Fig 6:**
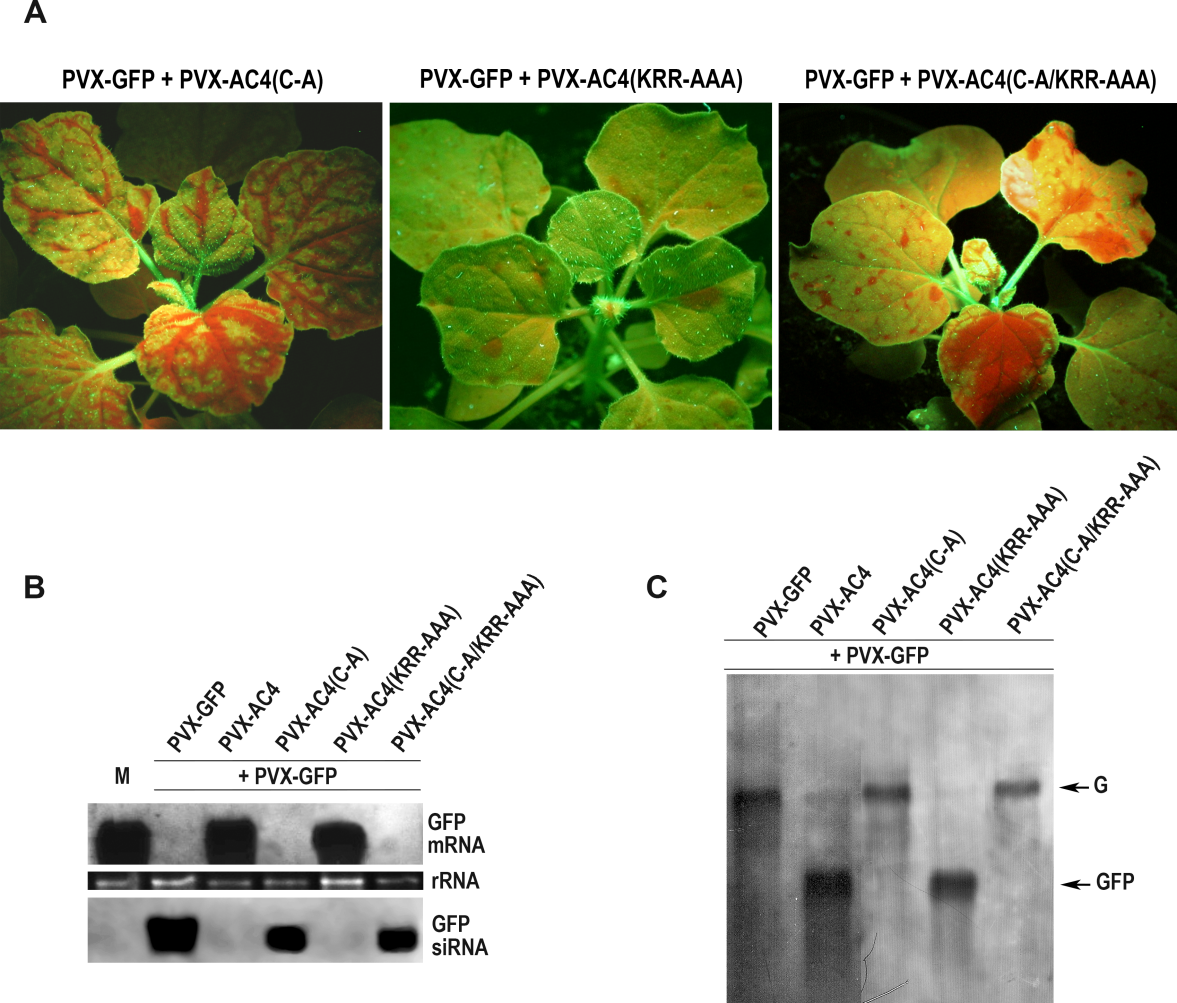
Effect of MYMV AC4 on PVX-induced GFP silencing. (A) Systemic spread of green fluorescence in *N*. *benthamiana* 16c plants agroinfiltrated with PVX-GFP plus PVX-AC4 mutants: PVX-AC4(C-A), PVX-AC4(KRR-AAA), and PVX-AC4(C-A/KRR-AAA) observed 15 days post infiltration. (B) Northern blot detection of GFP mRNA and siRNA from extracts of plants agroinfiltrated with PVX-AC4 and its mutants. (C) Northern blot detection of GFP mRNA from extracts of plants agroinfiltrated with PVX-AC4 and its mutants, showing a 3-fold shorter exposure of image (B). The same total RNA in the same amount was used for this and the Northern blot detection shown in Fig 3B. M, mock-agroinfiltrated plants. G, PVX-GFP genomic RNA; GFP, GFP mRNA. Constructs are shown at the top of the images. rRNA represents ethidium bromide-stained ribosomal RNA shown as loading control.

Starting from the onset of silencing, plants infiltrated with PVX-GFP in combination with the PVX-AC4(C-A) and PVX- AC4(C-A/KRR-AAA) showed the same systemic pattern as those infected with PVX-GFP alone (Fig 6A, compare with Fig 3A). On the other hand, similarly to PVX-GFP/PVX-AC4 (Fig 3A), plants inoculated with PVX-GFP/PVX-AC4(KRR-AAA) appeared fluorescent consistently with absence of GFP silencing (Fig 6A). From 12 dpi, only plants co-infected with PVX-AC4 or AC4(KRR-AAA) showed suppression of systemic silencing whereas plants inoculated with the C-mutated proteins, appeared red-fluorescent under UV light (Fig 6A). The GFP pattern remained unchanged even one month after agroinfiltration (S5 Fig).

RNA gel blot analysis confirmed that the persisting systemic expression of GFP in plants agroinfiltrated with PVX-GFP/PVX-AC4(KRR-AAA) was the result of inhibition of VIGS, and in turns that the AC4 NLS is dispensable for silencing suppression (Fig 6B and 6C). In fact, at 15 dpi the GFP siRNAs were detected in total RNA extracted from new leaves of plants agroinfiltrated with PVX-AC4(C-A) and PVX- AC4(C-A/KRR-AAA) but were absent in those infiltrated with PVX-AC4(KRR-AAA) (Fig 6B and 6C).

Consistently, GFP mRNA levels observed in plants co-inoculated with PVX-AC4 and PVX-AC4(KRR-AAA) were comparable with those in mock-inoculated plants (Fig 6B and 6C) confirming that the VSR function of AC4 is inhibited by mutation of C_11_.

These results strongly indicate that post-translational palmitoylation of AC4 and, consequent, PM localization are essential for efficient silencing suppression.

### AC4 interacts with the receptor-like kinase BAM1

**Fig 7:**
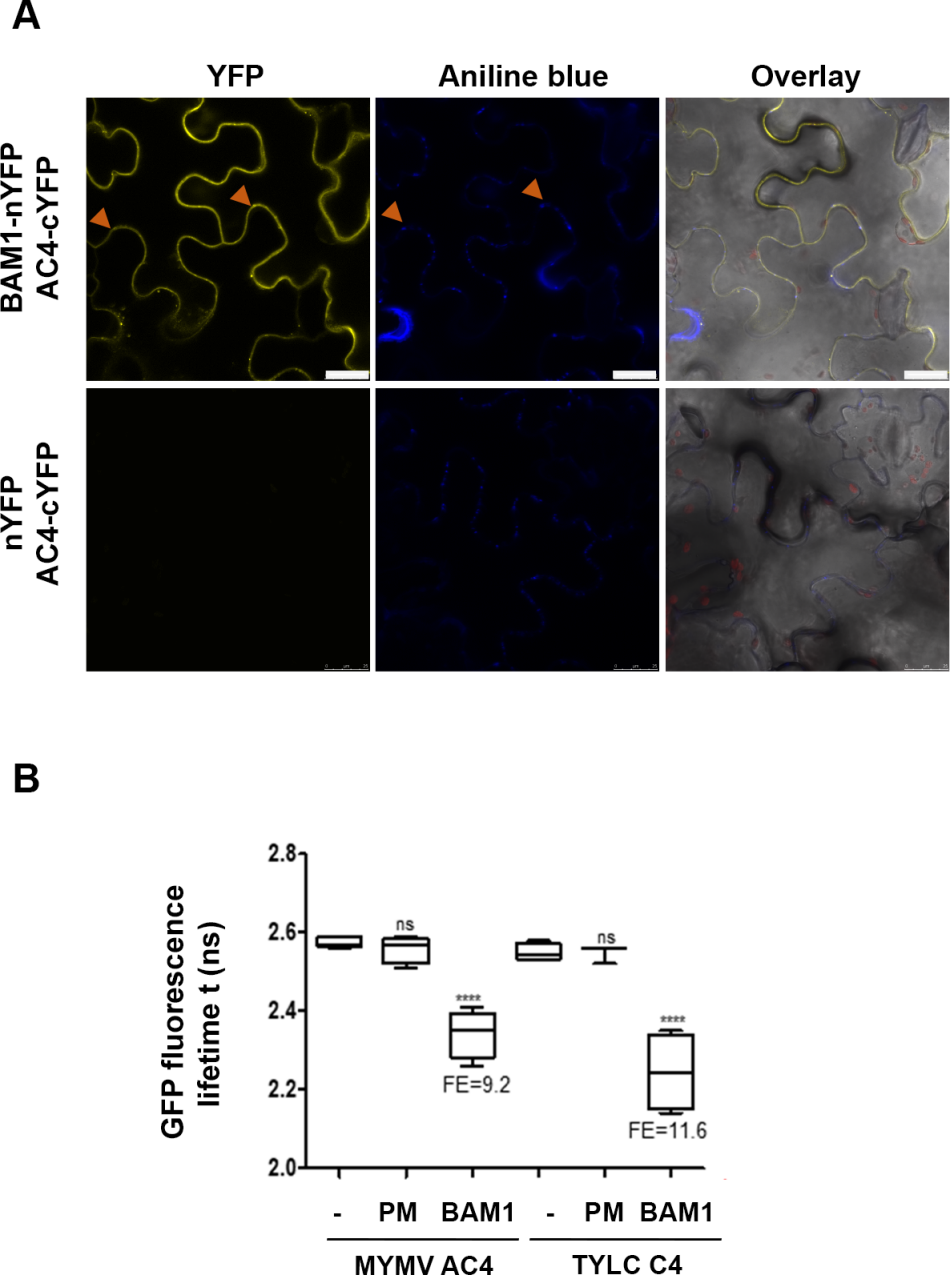
AC4 interacts with BAM1. (A) Interaction between AC4 and BAM1 by BiFC upon transient co-expression of the proteins n *N*. *benthamiana* leaves. BF: Bright field Arrowheads indicate plasmodesmata. Bars = 25 μm. (B) Interaction between AC4 and BAM1 by FRET-FLIM upon transient co-expression of the proteins in *N*. *benthamiana* leaves. The membrane protein NP_564431 (NCBI) is used as a negative control (PM control); the combination of C4 from TYLCV and BAM1 is used as a positive control. FE: FRET efficiency. Asterisks indicate a statistically significant difference (****,p-value < 0.0001; ns, no significant, according to a Student’s t-test.

Based on the evidence that the geminivirus TYLCV C4 targets PM and PD where interacts with BAM1 to inhibit intercellular spread of RNAi [36], we investigated whether MYMV AC4 could also interact with BAM1 in *N*. *benthamiana* leaves. Indeed, we observed that BAM1 and AC4 co-localize at PM and in PD (S6 Fig), and demonstrated their interaction by Bimolecular fluorescence complementation (BiFC) (Fig 7A). The interaction between AC4 and BAM1 was further confirmed using Fӧrster resonance energy transfer–fluorescence lifetime imaging (FRET-FLIM) (Fig 7B). These results convincingly support the evidence that AC4 requires PM localization for silencing suppression function.

### AC4 binds GFP-derived small RNAs

Collectively, our results indicate that AC4 undergoes a post-translational modification that mediates protein targeting to the PM. When localized to the PM, AC4 strongly suppresses systemic silencing whereas delocalization from such subcellular compartment impairs VSR activity. Furthermore, AC4 does not interfere with siRNA production and local PTGS and VIGS are not affected by the presence of the protein.

**Fig 8:**
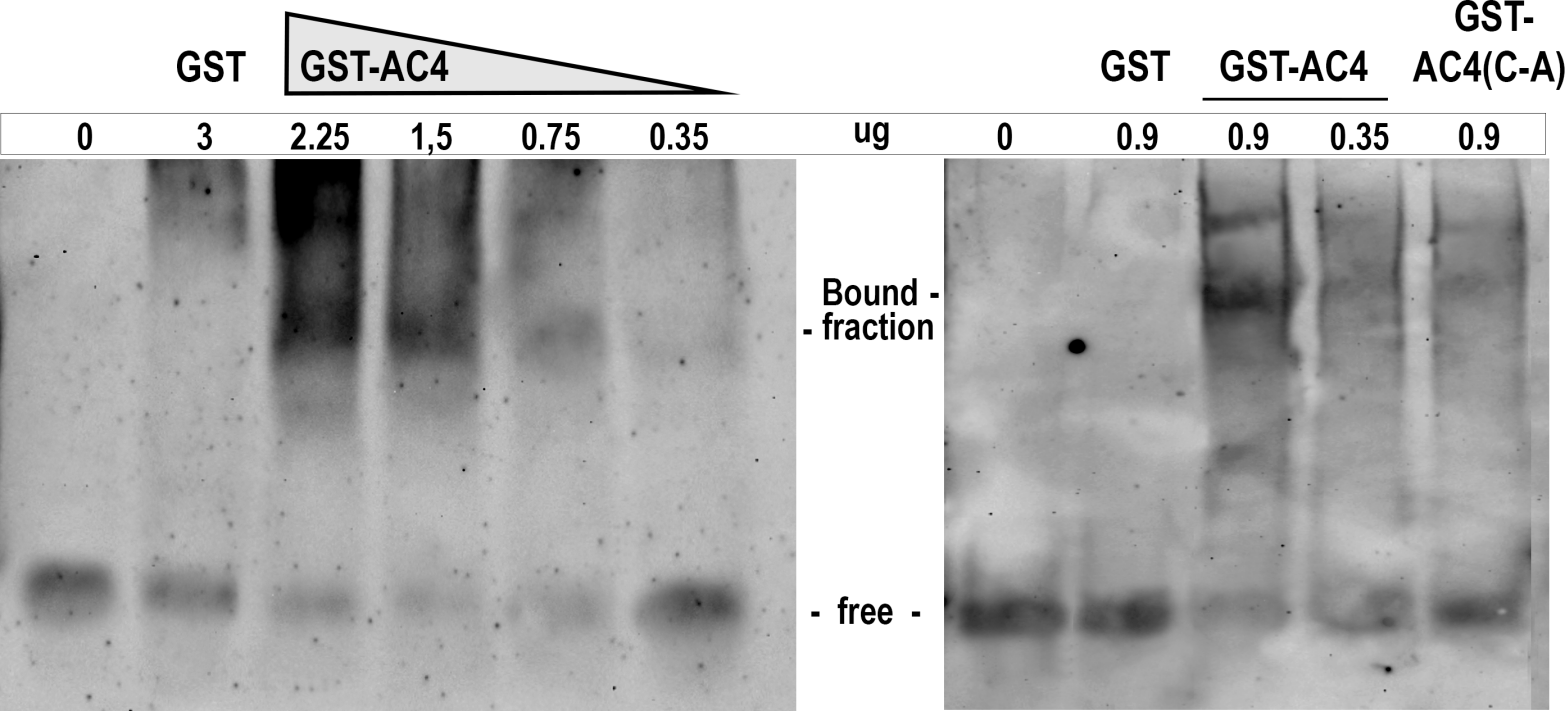
Gel mobility shift assay of AC4 binding to siRNA. PTGS-generated and gel-purified GFP siRNA (both gels, first lane) were used in a binding reaction with GST alone (both gels, second lane) and with increasing amount (shown above the gel) of GSTAC4. Binding reaction with the GSTAC4(C-A) mutant is shown in the last lane of the second gel. The position of protein-bound and–free siRNA fractions is shown on the gel side.

To investigate whether MYMV VSR might interfere with the transport of silencing signal by sequestering siRNA, we tested the ability of MYMV AC4 to bind small RNAs *in vitro* by electrophoretic mobility shift assay. For this assay, we used purified viral protein expressed in fusion with the glutathione S-transferase (GSTAC4) and gel purified GFP siRNAs produced upon PVX-GFP induced gene silencing in *N*. *benthamiana* 16c line. Upon combination with different concentration of GSTAC4, we observed slower migration of siRNAs indicating the formation of a protein-siRNA complex which confirmed the ability of MYMV AC4 to bind native 21–25 nt siRNAs (Fig 8). Such ability is not lost upon mutation of the palmitoylated C in A. In fact, the GSTAC4(C-A) mutant also binds siRNA, albeit probably less efficiently, as suggested by comparing the intensity of the siRNA fraction bound to equal amount of GSTAC4 and GSTAC4(C-A) (Fig 8, lanes 3 and 5 second gel).

## Discussion

AC4 is among the least conserved proteins of all geminiviruses and appears to have divergent biological functions among species of the family, being mostly involved in virus-plant interactions and in pathogenesis [48–51]. It was shown to play a role in the regulation of cell division [52] whereas, in other species, mutagenesis and/or transgenic expression of AC4 has no consequence on infection of several host plant [53].

Aiming at gaining insights into the mechanism of pathogenesis of MYMV and, more in detail, into the way of action of AC4, first and foremost, we have shown that infection of *V*. *mungo* with an AC4-deficient MYMV mutant develops asymptomatic phenotype, which reveals the essential role of AC4 for virus viability. Interestingly, this viral mutant replicates in inoculated leaves, albeit not at the same rate as the wild type MYMV and, opposite to MYMVΔBC1, lacking movement function, it moves short distance cell–to-cell whereas systemic transport is fully hindered.

Expression of MYMV AC4 increases severity of symptom induced by PVX in *N*. *benthamiana*, and suppresses systemic but not local GFP silencing in transgenic line 16c.

Interestingly, we observed that AC4 targets the PM few hours post inoculation but shortly after, it starts to accumulate also into the nucleus via a typical NLS, and such specific double localization is maintained in the course of infection.

The subcellular localization of AC4 is intriguing because normally, among plant viral proteins, only movement proteins localize to the PM. AC4 hosts no transmembrane domain but is post-translationally covalently modified by attachment of a lipid to the Cys_11_ that allows protein targeting and attachment to the PM. Such modification, known as S-acylation or palmitoylation and primarily meant to anchor otherwise soluble proteins to membranes, is now considered an important dynamic regulatory mechanism in signaling pathways in plants [25].

We found that the VSR activity of AC4 depends on protein binding to PM and is impaired upon mutation of Cys_11_. MYMV hosts another strong silencing suppressor: the transactivator AC2, which accumulates predominantly in the nucleus, excluding the nucleolus [12]. Opposite to AC2 that suppresses silencing in the nucleus, nuclear localization of AC4 is not related to this protein activity suggesting that the two MYMV VSR act in different cellular compartments and with different modalities. Therefore, the significance of AC4 targeting the nucleus remains to be investigated. Interestingly, we observed that PM is the first localization of MYMV AC4 while nuclear accumulation is visible later after transfection. This suggests that the protein could target the nucleus when accumulation to the PM attains saturation in a cycle of natural turnover for palmitoylated proteins [54, 55]. Alternatively, activation/inhibition of palmitoylation could be a strategy to switch between different protein functions requiring distinct subcellular localization [30]. In fact, considering that some viral proteins evolved silencing suppression activity after or concomitantly with other functions essential for virus viability [9], another role distinct from silencing suppression, which we proved to be uncoupled from nuclear localization, cannot be ruled out for AC4.

The absolute requirement of the Cys_11_ for silencing suppression activity reflects the need of such specific localization and justifies the highly stable association of AC4 to PM. The VSR function of *East African cassava mosaic virus* (EACMV) AC4 is also dependent on localization to the PM [18]. This protein is predicted to be N-myristoylated, and this modification is correlated to the VSR function. Proteins with the potential to become S-acylated often undergo myristoylation to interact with membranes and, subsequently, they become S-acylated and fixed to them [56]. Interestingly, EACMV AC4 hosts also a Cys in a favorable context for palmitoylation, whose mutation partially restricts the protein on perinuclear vesicles [18]. While palmitoylation of MYMV AC4 helps both binding and docking of the protein to the PM, in EACMV AC4, the two actions could be mediated by palmitoylation and myristoylation, respectively. However, as the authors did not confirm experimentally the post-translational modification of the protein, it remains to be demonstrated the role of palmitoylation for the AC4 of this begomovirus.

In *Begomovirus* infecting cassava, such as EACMV, *African cassava mosaic virus* (ACMV) and *Indian cassava mosaic virus* (ICMV), both AC2 and AC4 VSR are functional and matched in a way that when AC2 is a strong suppressor its correspondent AC4 is a mild suppressor, and vice-versa [38].

Conversely, MYMV AC2 and AC4 are both strong VSR with distinct subcellular localization, and apparently both essential. The evidence that, despite the presence of functional AC2, MYMV-ΔAC4 failed to establish systemic infection in *V*. *mungo* confirms that AC2 cannot compensate AC4 VSR function, and vice-versa [12].

While several examples of siRNA-sequestering VSRs as well as some movement proteins also acting as silencing suppressors are described in the literature [9], to our best knowledge this is the first report of a siRNAs-binding VSR that absolutely requires PM localization to perform its function.

AC4 is not a movement protein and the absolute requirement of PM localization for its silencing suppression activity is very interesting. TYLCV C4 also targets the PM and binds to BAM1 to hinder the spread of silencing signal triggered by this receptor-like kinase [36]. We demonstrate that MYMV AC4 also binds BAM1 and similar to C4 might hinder the silencing-related function of BAM1. However, based on the experimental evidences collected in this study and particularly the capacity of MYMV AC4 (unknown for TYLCV C4) to bind siRNAs and its much stronger VSR ability compared to C4 [57], we hypothesize a different or additional mechanism of action for this protein. Upon targeting to the PM and particularly to PD, MYMV AC4 could bind siRNAs and stop their passage to the neighbouring cell thus suppressing the spread of the PTGS signal through the plant. However, further experimental *in vivo* evidence required to confirm this working hypothesis.

The two nonpolar phenylalanines flanking Cys_11_, might have the important role of creating the required hydrophobic environment to allow association of the hydrophilic AC4 to the PM and the insertion of palmitate into the double lipid layer of PM [47]. This specific amino acid context (Phe-Cys-Phe) suggests that AC4 could dock to the PM folded in shape of “V” where the bottom tip is occupied by the cysteine bond to palmitic acid inserted into PM. The two phenylalanines would provide hydrophobic stability to the bond whereas the hydrophilic tails of AC4 would be kept on the cytoplasmic side, away from the membrane and available for interactions with siRNAs. The evidence that the AC4(C-A) mutant, missing the palmitoylated C, binds siRNA less stronger than AC4 WT, supports the hypothesis that the “Phe-Cys-Phe” hydrophobic domain might be important for conformational stability of the protein.

Whether the AC4 interaction with BAM1 is functional in regulation of RNAi cell-to-cell spreading, and if this strategy is complementary or synergic with the siRNA sequestering capacity of AC4 is yet to be elucidated.

Antiviral systemic signaling is a still unknown aspect of host defense and further validation is required to prove that plant immunity can be reached by systemic movement of vsiRNA. However, taken together the evidence here provided, it is tempting to speculate that MYMV AC4 would hijack the host lipidation machinery to target PD and, by binding vsiRNA, block the signal of “plant immunity”.

The existence of different types of geminiviral VSRs, suggests that these proteins (co)-evolved to target different steps of the silencing pathway in a temporal and/or spatial manner. In the case of MYMV for example, AC4 might strategically be localized to PD to pose a physical barrier to the spread of silencing signal that could have escaped the suppression control of AC2. As several suppressor proteins have multiple roles, including non-silencing functions critical for virus viability, and their synchronized action is essential in order to fulfill the multiple tasks, post translational modification could be an efficient strategy to reach this goal. In fact, S-acylation and/or N-myristoylation is predicted in AC4/C4 of the *Geminiviridae* species (S2 Table), and phosphorylation, another mechanisms of post-translational modification, has been recently reported to regulate subcellular localization and in turn VSR activity of cucumber mosaic virus 2b protein [58]. Therefore, post-translational modification and its correlation to VSR function should be considered and investigated, particularly for those proteins with multifunctional behavior and potential localization to membrane compartment.

In this study, we present the first report of palmitoylation and more in general of lipidation of a plant viral protein. The critical role of this post-translational modification on the function of MYMV AC4 suggests that lipidation is a very reliable way to target viral proteins to the membrane compartment and that more viral proteins might use these modifications for regulating their function at membranes.

## Materials and methods

### Cloning and expression of AC4 and mutants

All plasmids used in transient expression experiments are based on pCKGFP [59], modified by replacing GFP with the EGFP coding sequence and by the addition of two restriction sites (*Mlu*I and *Xba*I) at the 3' end.

The AC4 wt ORF was amplified by PCR from the pGA1.3A clone [37] with AC4-F and AC4-R primers (S1 Table) and the product was cloned into the *Mlu*I-*Xba*I sites of pCKEGFP in frame with EGFP. AC4 (C11-A) and AC4 (KRR-AAA) mutants were derived from pCKEGFPAC4wt using the QuickChange XL Site-Directed Mutagenesis Kit (Agilent Technologies) with AC4(C11-A)F/AC4(C11-A)R and AC4(KRR-AAA)F/AC4(KRR-AAA)R pairs of primers covering the aminoacids mutated, according to the manufacturer’s instructions. Plasmids containing mutated C and KRR were used as a template for site-directed mutagenesis to obtain the pCKEGFPAC4 (C11-A /KRR-AAA).

The AC4 sequence encoding aminoacids 1 through 12 was fused in frame with the 5’ of EGFP by amplifying EGFP with a forward primer containing the AC4 sequence fragment.

Infectious clones based on the PVX genome, were obtained from the pGR107 plasmid. AC4 wt and mutant ORFs were amplified from the pCKEGFP clones with AC4(SmaI)F and AC4(SalI)R (S1 Table) whereas the p19 and the mGFP5 sequences were amplified from the pGA482p19 clone [41] with p19SmaI and p19SalI primers (S1 Table) and from DNA extracted from *N*. *benthamiana* 16c plants with mGFP5_F(SmaI) and mGFP5_R(SalI) primers (S1 Table), respectively. PCR products were cloned in the *Sma*I-*Sal*I sites of the multiple coning site of the pGR107 plasmid and the obtained plasmids introduced into *Agrobacterium tumefaciens* strain C58C1 by a freeze–thaw method.

AC1, AC4 and BC1 genes were knocked-out within the virus infectious clones pGA1.3A (DNA A wt) and pGA1.3B (DNA B wt) [37] using the QuickChange XL Site-Directed Mutagenesis Kit (Agilent Technologies) and the MYMV-AC1koF/ MYMV-AC1koR, MYMV-AC4koF/MYMV-AC4koR, and MYMV-BC1koF/MYMV-BC1koR pairs of primers (S1 Table) covering the aminoacids mutated, respectively.

For expression and suppression of GFP RNA silencing, the binary plasmid pGA482p19 and a pCAMBIAAC4 plasmid based on pCAMBIA32 modified by cloning an expression cassette containing AC4 under the control of the 35S promoter in *Pst*I restriction site, were introduced into *Agrobacterium tumefaciens* strain C58C1 by a freeze–thaw method.

For the electrophoretic mobility shift assay, MYMV AC4 was amplified with the MYMVAC4 F/ MYMVAC4-HA R pair of primers (S1 Table) and cloned into the EcoRI/SalI sites of pGEX-6p-1 (GE Healthcare) in frame with the glutathione S-transferase (GST) coding sequence. The MYMVAC4-HA R reverse primer (S1 Table) contained the sequence encoding the 9 aminoacids of the HA epitope (TAC CCA TAT GAC GTC CCA GAT TAC GCT encoding YPYDVPDYA).

The third stop codon following the SalI site in the pGEX6p1 plasmid in frame with AC4 sequence was used for termination of translation. The HA (human influenza hemagglutinin) epitope tag was engineered onto the C- terminus of AC4 sequence so that the tagged protein could be analyzed and visualized using immunochemical methods.

For BiFC and FRET-FLIM analysis, AC4 was cloned in pENTRD/TOPO (Invitrogen) using primers CACCATGAAGATGGAGAACCTCATCT and GTATATTGAGGGCCTGTAACTTG. Gateway cloning (Invitrogen) was used to fuse AC4 to GFP in pGWB505 [60], to RFP in pB7RWG2.0 [61], and nYFP/cYFP in pGTQL1211YN/pGTQL1221YC [62].

Construct to express C4-GFP, BAM1-RFP, PM-RFP (Plasma Membrane protein NCBI number NP_564431), C4-cYFP, BAM1-cYFP and BAM1-nYFP are described in Rosas-Diaz et al., 2018.

### Protoplast and plant transfection and fluorescence microscopy analysis

Protoplasts were isolated from *N*. *benthamiana* and transfected as described [63]. *V*. *mungo* plants were biolistically transfected with clones to express AC4 WT and mutants and stained with DAPI. Fluorescent proteins were examined with a Nikon Eclipse 80i microscope equipped with video confocal technology (VICO). For GFP, DsRed and DAPI images, the ET-GFP filter set (Chroma 49002, Nikon), the G-2A filter (Nikon) and DAPI filter were used, respectively.

*N*. *benthamiana* plants were agroinfiltrated with clones to express BAM1-GFP and AC4-RFP and stained with aniline blue. Imaging was performed as described in Rosas-Diaz et al., 2018.

### Bimolecular fluorescent complementation and FRET-FLIM imaging

Bimolecular fluorescent complementation (BiFC) assays were performed as described previously [36]. In brief, *N*. *benthamiana* plants were agroinfiltrated with clones to express the corresponding proteins, and samples were imaged two days later on a Leica TCS SMD FLCS confocal microscope, using the pre-set settings for YFP with Ex:514 nm, Em: 525–575 nm.

FRET-FLIM experiments were performed as described previously [36].

### Agroinfiltration and GFP imaging

*N*. *benthamiana* plants were grown at 25°C. At six-leaves stage plants were infiltrated with *A*. *tumefaciens* C58C1 harboring the appropriate constructs. *A*. *tumefaciens* carrying each construct was grown on selective media overnight, resuspended in the infiltration buffer (10 mM MES, 0.15 mM acetosyringone, 10 mM MgCl), kept at 25°C for 2-3h, and subsequently infiltrated into wt or 16c plant leaves at OD = 1. In co-infiltration experiments, equal volumes/concentration of each suspension were mixed prior to infiltration. GFP fluorescence was observed under long-wavelength UV light (Black Ray model B 100A, UV Products) and photographed with a yellow filter.

### Real time assay and statistical analysis

Leaf samples from MYMV bombarded *V*. *mungo* plants were collected and analysed in triplicates. DNA was extracted by Dellaporta method [64] with slight modification. 50 mg fresh leaf tissue was ground in liquid nitrogen, mixed with extraction buffer (50 mM Tris-HCl pH 8.0, 20 mM EDTA pH 8, 350 mM NaCl, 8 M Urea, 2% N-Lauril-Sarcosine) and equal volume of phenol and incubated at 70°C for 5 min. DNA was extracted from the supernatant upon centrifugation by volume of phenol: chloroform (1:1), isopropanol precipitation and RNase treatment.

Two different couples of primers were designed: VrACtfor/ VrACtrev to amplify an endogenous actin gene (*Vigna radiata* actin, accession number AF143208) and AC2-RT_F/ AC2-RT_R to amplify the target MYMV AC2 gene (S1 Table).

Relative qPCR was performed using C1000 thermal cycler (Bio-Rad). The cycling profile consisted of 95°C for 20 s, 40 cycles of 3 s at 95°C and 30 s at 60°C, one cycle of 10 s at 95°C, as recommended by the manufacturer, using 2X Fast SYBR Green PCR Master Mix (Applied Biosystems), 400 nM forward and reverse primers, 4 ng of *V*. *mungo* DNA and nuclease-free water in a total volume of 12.5 μL.

Each DNA sample was amplified in duplicate for each primer pair and immediately after the final PCR cycle, a melting curve analysis was performed to determine the specificity of the reaction. Relative quantification was calculated using the comparative cycle threshold (Ct) method (RQ = 2^–ΔΔCt^) [65], in which the change in the amount of the target viral RNA was normalized in relation to the endogenous control.

Data were log-transformed [log(*x*+1)] before statistical analysis in order to fulfil the assumptions for parametric statistics. Transformed data were analyzed in a repeated measure factorial design using the MIXED procedure of SAS (SAS/Stat Inc.), in which the variables ‘Experiment’, ‘Treatment’ and ‘Time’ were considered as fixed effects. Contrasts were performed to test the experiment reproducibility, *viz*. the hypothesis of no difference between the two independent experiments made, and the difference among treatments.

### RNA analysis

Total RNA was extracted from 100 mg of leaf tissue. Plant materials homogenized in liquid nitrogen was resuspended in 600 μl of extraction buffer (0.1 M Glycine-NaOH, pH 9.0, 100 mM NaCl, 10 mM EDTA, 2% SDS) and mixed with an equal volume of phenol. The aqueous phase was treated with equal volumes of phenol-chloroform, precipitated with ethanol, and finally resuspended in sterile water. RNA gel blot analysis of higher molecular weight RNAs was performed as previously described [41]. For analysis of siRNAs, low-molecular-weight RNAs (LMW-RNAs) were enriched from total RNAs extract by removing high-molecular-mass RNAs with 10 % polyethylene glycol (PEG8000) and 1M NaCl. Approximately 5 μg of LMW RNAs were separated by 17 % PAGE with 7 M urea and then blotted onto Hybond-N+ membranes. After UV cross-linking, the membranes were hybridized at 42°C and the detection was carried out with DIG non-radioactive system (Roche Applied Science) according to the manufacturer’s instructions using a probe covering the entire mGFP5 sequence (GenBank: U87973). The blots were incubated in antibody solution, anti-DIG-AP Conjugate (Roche) and CDP-STAR (Roche) for chemiluminescence detection.

### Protein extraction from protoplasts

Cell fractionation was performed as previously described with some modifications [66] [67]. Protoplasts transfected with pCKAC4HA plasmid were pelleted, resuspended in buffer (1X PBS (pH 7.4), protease inhibitors, 0.5% Tween) and lysed by 5 freeze-and-thaw cycles. Cell debris (P_0.5_) was isolated by 3 min centrifugation at 500 x g, 4°C. The supernatant (S_0.5_) was further centrifuged at 30,000 x *g* and 4°C for 30 min to yield supernatant (S_30_) and pellet fractions (P_30_).

For analysis of the membrane part, the P30 fraction was incubated for 30 min on ice in the presence of one of the following reagents: 100 mM Na_2_CO_3_ (pH 11.5), 4 M urea, or 1 M KCl [68]. After centrifugation at 30,000 x *g* for 30 min at 4°C, pellets and supernatants were resolved on 12% SDS-PAGE, transferred to Hybond PVDF membrane (Millipore) and subjected to Western blot analysis. Proteins were detected with anti HA antibody (1:4000, Santa Cruz Technology), visualized with SuperSignal West Pico Chemiluminescent Substrate (Pierce) according to the manufacturer’s instructions and scanned by Chemidoc Touch Imaging System (BioRad).

### Biotin switch assay

For biotin switch assay, collected protoplasts transfected with pCKAC4HA and pCKAC4(C-A)HA were resuspended in 500 μL lysis buffer (1X PBS pH 7.4, protease inhibitors, 1 mM EDTA, 1% Triton X-100, 25 mM N-ethylmaleimide) and treated following the method described [69]. Eluted proteins were analyzed by SDS-PAGE and Western blotting as described above.

### Protein expression and purification for RNA binding

Recombinant GSTAC4, GSTAC4(C-A), and GST proteins were produced by overexpression in *Escherichia coli* BL21 codon plus cells (Agilent Technologies). Cells were grown to OD_600_ ≈ 0.7 and IPTG was added to a final concentration of 0.4 mM for AC4 and of 0.2 mM for GST for induction (3 h at 37°C). GST fusion protein supernatants obtained after bacterial lysis and centrifugation were purified with Glutathione Sepharose 4B beads (GE Healthcare) following the manufacturer’s instructions.

### RNA-protein binding

For preparation of 21-25-nucleotide siRNA, 45 to 50 μg of total RNA were electrophoresed on 17% PAGE with 8 M urea followed by ethidium bromide staining in 1 × Tris-borate-EDTA. 21–25 nt siRNA fraction was cut and incubated in buffer 0.3 M NaCl, 0.1% SDS, overnight at 4°C with rocking. After a gentle centrifugation for 5 min at 2000g, the supernatant was transferred to a 50 mL tube. The crushed gel slice was incubated for a second elution in the same buffer with rocking. The gel residues were pelleted by centrifugation, and the two supernatants were precipitated together with ethanol.

For binding assays, increasing amount of purified GSTAC4 and of GSTAC4(C-A), and siRNA (1.5 ug) were mixed and incubated for 20 min at room temperature in binding buffer (20 mM Tris–HCl pH 8, 5 mM MgCl_2_, 50 mM KCl, 25 mM NaCl and 2.5 mM DTT, 0.02% Tween, 10% glycerol). Each sample contained 40U RNasin. The reaction was stopped by adding dyes, and loaded onto 8% native PAGE. The gel was transferred to Hybond-N+ membrane and after UV cross-linking, the membranes were hybridized as described above.

## Supporting information

S1 Fig Quantification of MYMV accumulation by qPCR of AC2 gene from 1 through five days after *V*. *mungo* inoculation. Each value is the mean of three biological replicates and vertical bars indicate standard errors. RQ are normalized to the amount of plant DNA, represented by the endogenous actin gene. The statistical significance of values of expression between the samples at the same time point is the result of the analysis of variance. Differences were assumed to be statistically significant, and indicated with different letters from a, highest difference to c, lowest difference, for P < 0.01 (Duncan’s test).
(TIF)

S2 Fig Subcellular localization of AC4 and its mutant variants in *V*. *mungo* cells. Transient expression of GFP-fused wild type AC4 (GFPAC4) and its mutant variants (C-A, KRR-AAA) in mesophyll of bombarded *V*. *mungo* leaves. Filtered fluorescence images of GFP-AC4 and mutants (right column), DAPI-stained nuclear DNA (second row). If GFP-AC4 (green) is localized to the nucleus (dark blue), the latter appears light blue in the merge images (right row). Bars = 20 **μ**m.
(TIF)

S3 Fig Localization of GFP-AC4 in plasmodesmata. Coexpression of GFP-AC4 with the PD marker TMV DsRed-MP. Overlay of images shows colocalization to PD. Bars = 10 μm.
(TIF)

S4 Fig Structural and chemical prediction of AC4. Plot of peptide structure of AC4 obtained from the web-interface SeqWeb of GCG Wisconsin Package. Green line shows hydrophilicity probability, purple line illustrates the amino acid probability to be exposed at the protein surface. Palmitoylated cysteine and NLS (KRR) are indicated by arrows.
(TIF)

S5 Fig Late effect of MYMV AC4 on PVX-induced GFP silencing. Systemic spread of green fluorescence in *N*. *benthamiana* 16c plants agroinfiltrated with PVX-GFP plus PVX-AC4 mutants observed 30 days post infiltration.
(TIF)

S6 Fig Co-localization of MYMV AC4 and BAM1 at PM and PD. Subcellular co-localization of BAM1-GFP and AC4-RFP upon transient co-expression in *N*. *benthamiana* leaves two days post infiltration. BF: Bright field. Arrowheads indicate plasmodesmata. Bars = 25 μm.
(TIF)

S1 Table List of primers used for constructs preparation. Bases in bold show mutated bases. Bases underlined correspond to specific restriction enzyme sequences. Bases in italic in the MYMVAC4-HA R primer, correspond to the HA epitope.
(DOCX)

S2 Table Partial list of representative *Geminiviridae* AC4/C4 N-terminus sequence. Highlighted are predicted N-myristoylated (blue) and Palmitoylated (red) amino acids.
(DOC)
