# Supplementary figures and images for: S‐acylation mediates Mungbean yellow mosaic virus AC4 localization to the plasma membrane and in turns gene silencing suppression
Source: PLoS Pathog. 2018 Aug 1;14(8):e1007207. doi: 10.1371/journal.ppat.1007207 (PMC6089456; doi:10.1371/journal.ppat.1007207)

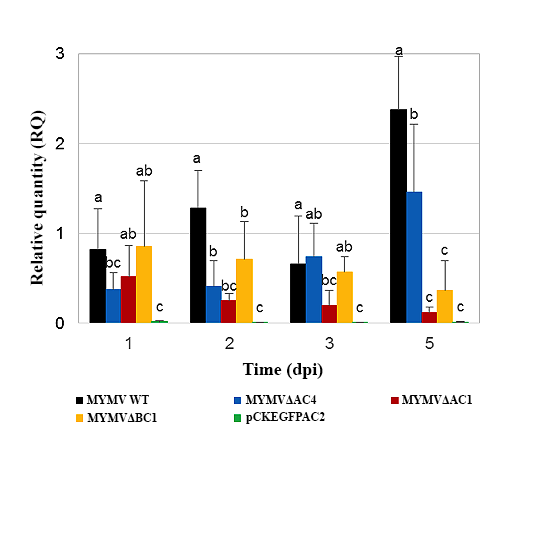

Supplement: S1 Fig — Each value is the mean of three biological replicates and vertical bars indicate standard errors. RQ are normalized to the amount of plant DNA, represented by the endogenous actin gene. The statistical significance of values of expression between the samples at the same time point is the result of the analysis of variance. Differences were assumed to be statistically significant, and indicated with different letters from a, highest difference to c, lowest difference, for P < 0.01 (Duncan’s test). (TIF) [file ppat.1007207.s001.tif]

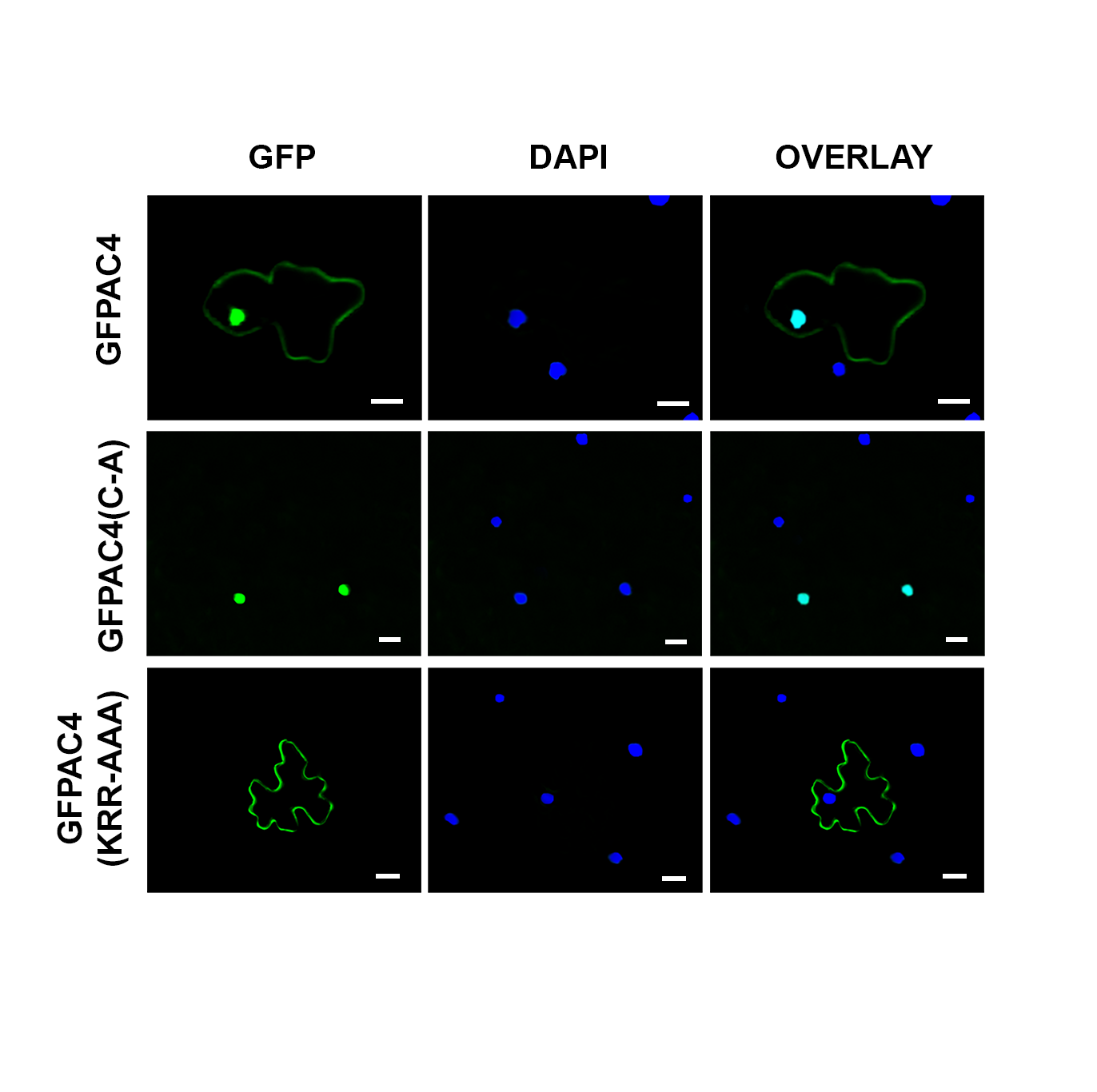

Supplement: S2 Fig — Transient expression of GFP-fused wild type AC4 (GFPAC4) and its mutant variants (C-A, KRR-AAA) in mesophyll of bombarded V. mungo leaves. Filtered fluorescence images of GFP-AC4 and mutants (right column), DAPI-stained nuclear DNA (second row). If GFP-AC4 (green) is localized to the nucleus (dark blue), the latter appears light blue in the merge images (right row). Bars = 20 μm. (TIF) [file ppat.1007207.s002.tif]

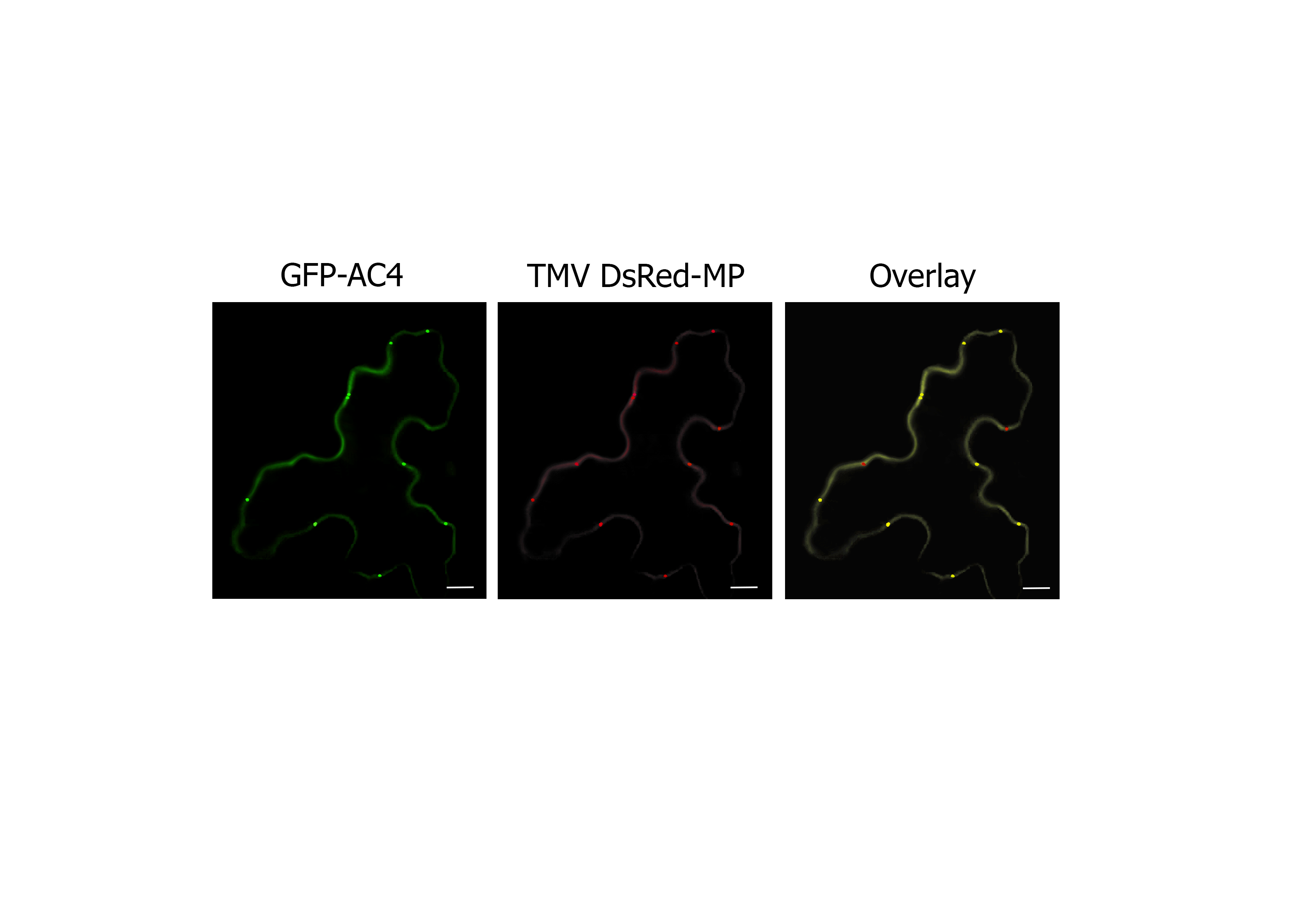

Supplement: S3 Fig — Coexpression of GFP-AC4 with the PD marker TMV DsRed-MP. Overlay of images shows colocalization to PD. Bars = 10 μm. (TIF) [file ppat.1007207.s003.tif]

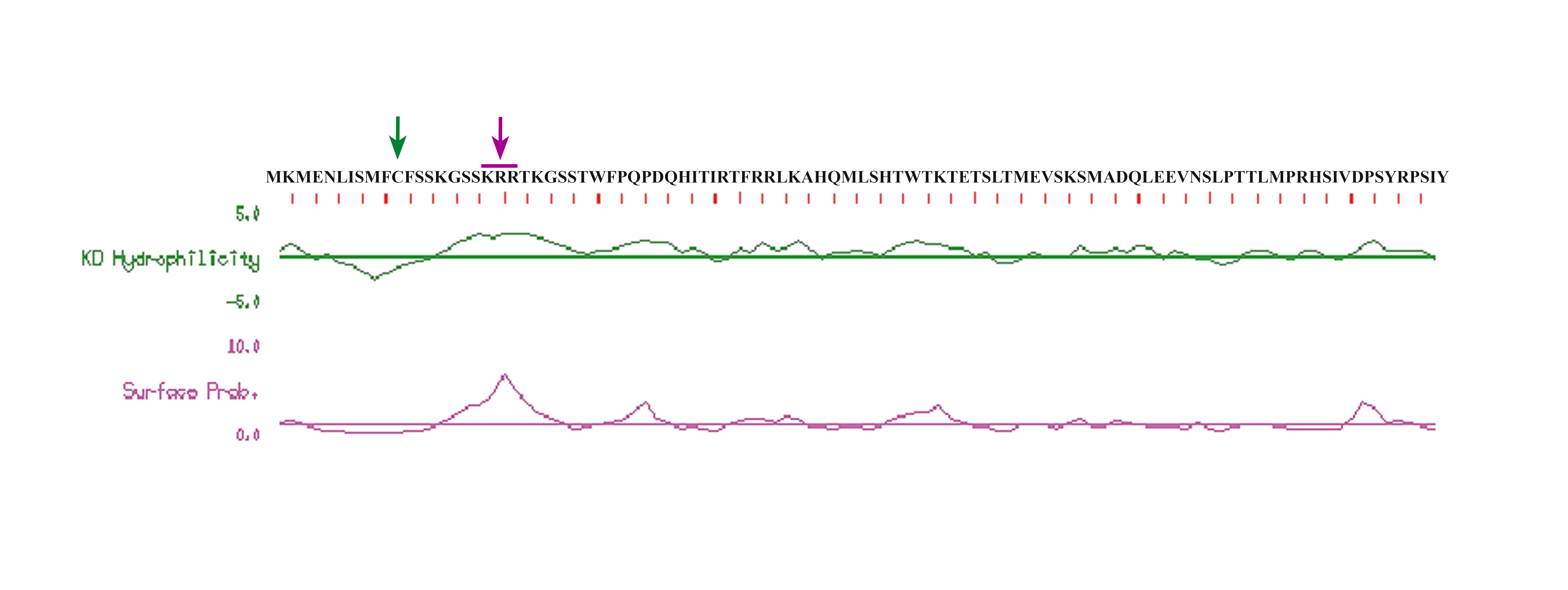

Supplement: S4 Fig — Plot of peptide structure of AC4 obtained from the web-interface SeqWeb of GCG Wisconsin Package. Green line shows hydrophilicity probability, purple line illustrates the amino acid probability to be exposed at the protein surface. Palmitoylated cysteine and NLS (KRR) are indicated by arrows. (TIF) [file ppat.1007207.s004.tif]

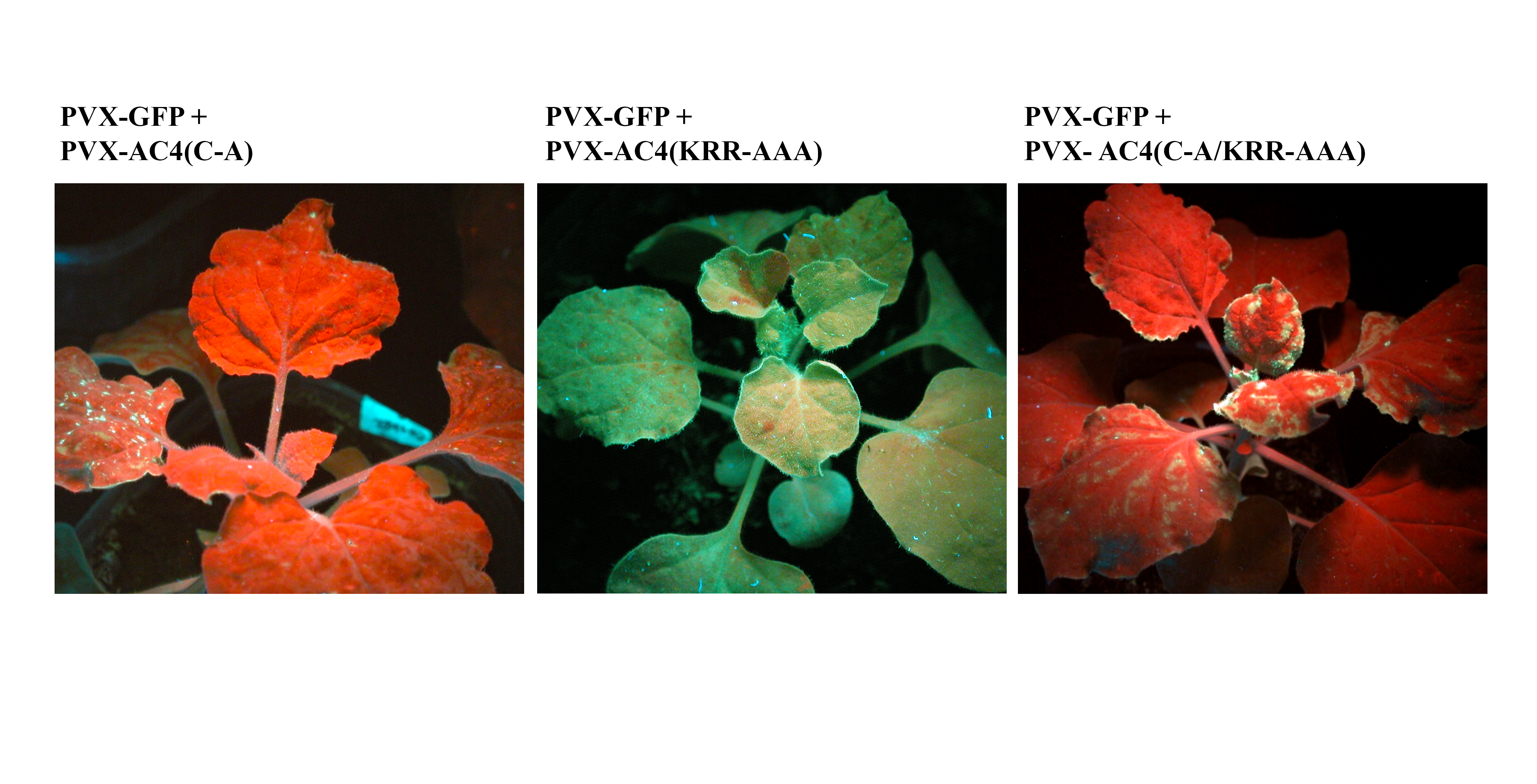

Supplement: S5 Fig — Systemic spread of green fluorescence in N. benthamiana 16c plants agroinfiltrated with PVX-GFP plus PVX-AC4 mutants observed 30 days post infiltration. (TIF) [file ppat.1007207.s005.tif]

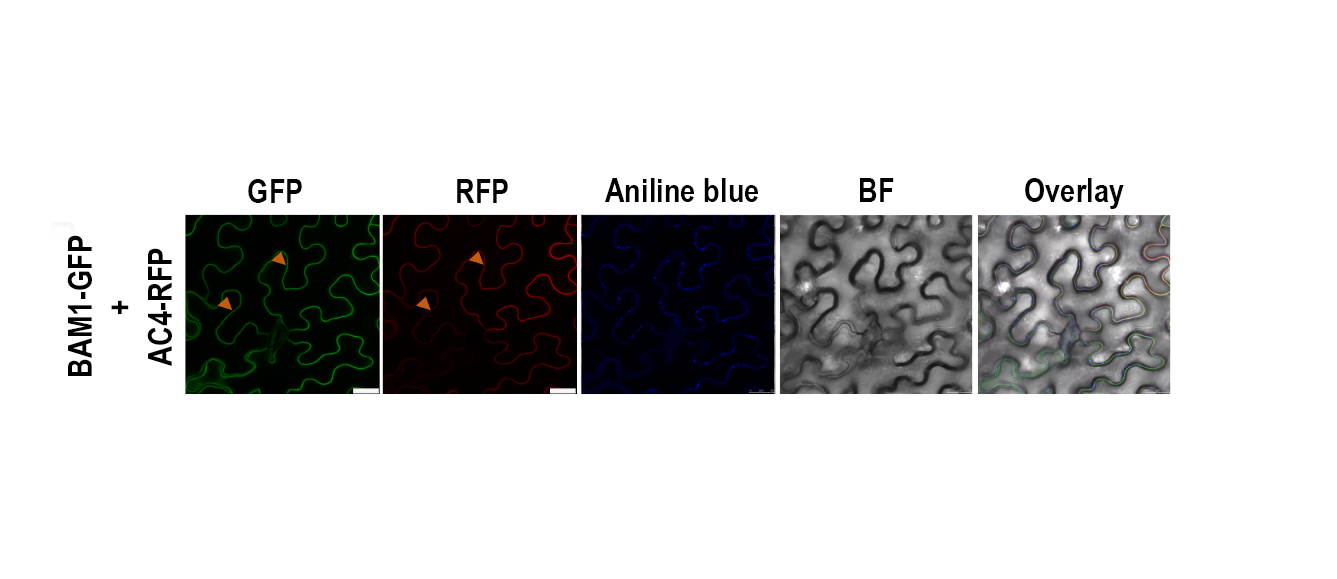

Supplement: S6 Fig — Subcellular co-localization of BAM1-GFP and AC4-RFP upon transient co-expression in N. benthamiana leaves two days post infiltration. BF: Bright field. Arrowheads indicate plasmodesmata. Bars = 25 μm. (TIF) [file ppat.1007207.s006.tif]
