# Supplementary material for: S‐acylation mediates Mungbean yellow mosaic virus AC4 localization to the plasma membrane and in turns gene silencing suppression
Source: PLoS Pathog. 2018 Aug 1;14(8):e1007207. doi: 10.1371/journal.ppat.1007207 (PMC6089456; doi:10.1371/journal.ppat.1007207)
Supplement: S1 Table — Bases in bold show mutated bases. Bases underlined correspond to specific restriction enzyme sequences. Bases in italic in the MYMVAC4-HA R primer, correspond to the HA epitope. (DOCX) [file ppat.1007207.s007.docx]

S1 Table.

List of primers used for constructs preparation. Bases in bold show mutated bases. Bases underlined correspond to specific restriction enzyme sequences. Bases in italic in the MYMVAC4-HA R primer, correspond to the HA epitope.

| AC4-F | CGACGCGTGAAGATGGAGAACC |
| --- | --- |
| AC4-R | CGTCTAGATCAGTATATTGAGGG |
| AC4(C11-A)F | CCTCATCTCCATGTTC**GC**CTTCAGTTCGAAGGG |
| AC4(C11-A)R | CCCTTCGAACTGAAG**GC**GAACATGGAGATGAGG |
| AC4 (KRR-AAA)F | CGAAGGGAAGCTCC**GCAGCAGCA**ACGAAAGGTTCTTCG |
| AC4 (KRR-AAA)R | CGAAGAACCTTTCGT**TGCTGCTGC**GGAGCTTCCCTTCG |
| AC4(SmaI)F | tccCCCGGGatgaagatggagaacctcatctcc |
| AC4(SalI)R | acgcGTCGACtcagtatattgagggcc |
| mGFP5_F(SmaI) | tccCCCGGGatgagtaaaggagaagaacttttcactgg |
| mGFP5_R(SalI) | ACGCGTcgacttatttgtatagttcatccatgcc |
| p19SmaI | tccCCCGGGatggaacgagctatacaagg |
| p19SalI | ACGCGTcgacttactcgctttctttcttga |
| MYMV-AC4koF | CGTGAACTCCA**C**GAAGA**C**GGAGAACCTCATCTCCA**C**GTTCTGCTTCAGTTCG |
| MYMV-AC4koR | CGAACTGAAGCAGAAC**G**TGGAGATGAGGTTCTCC**G**TCTTC**G**TGGAGTTCACG |
| MYMV-AC1koF | CAAAACGACGCCAAAATAT**A**CCTAGACTCGGTCG |
| MYMV-AC1koR | CGACCGAGTCTAGG**T**ATATTTTGGCGTCGTTTTG |
| MYMV-BC1koF | GCAACACAATTTCTGAATA**AT**GAGAATTATTCAGGCGC |
| MYMV-BC1koR | GCGCCTGAATAATTCTC**AT**TATTCAGAAATTGTGTTGC |
| MYMVAC4 F | cGAATTCaagatggagaacctc |
| MYMVAC4-HA R | actcGTCGAC*AGCGTAATCTGGGACGTCATATGGGTA*gtatattgagggc |
| VrACtfor | gcattggcaccgagtagcatgaaa |
| VrACtrev | atctgttggaaggtgctgagggaa |
| AC2-RT_F | tgcgctcctcagcgcttacataat |
| AC2-RT_R | agtcgttcaccaatggatcccaca |
