# Supplementary material for: S‐acylation mediates Mungbean yellow mosaic virus AC4 localization to the plasma membrane and in turns gene silencing suppression
Source: PLoS Pathog. 2018 Aug 1;14(8):e1007207. doi: 10.1371/journal.ppat.1007207 (PMC6089456; doi:10.1371/journal.ppat.1007207)
Supplement: S2 Table — Highlighted are predicted N-myristoylated (blue) and Palmitoylated (red) amino acids. (DOC) [file ppat.1007207.s008.doc]

**S2 Table. Partial ist of representative *Geminiviridae* AC4/C4 N-terminus sequence.** Highlighted are predicted N-myristoylated (blue) and Palmitoylated (red) amino acids.

**Accession**

**number Begomovirus isolate N-terminus sequence**

P0C6F9 ABMVW *Abutilon mosaic virus* MCSYSSKANTNARITDSSIWSPQPGQHISI----

Q91J24 BCTVC *Beet curly top virus* MGNLISTSCFNSKEKFRSQISDYSTWYPQP----

Q9YPS1 MYMVV *Mungbean yellow mosaic virus*  MKMENLISMFCFSSKGSSKRRTKGSSTWFP----

Q88891 TPCTV *Tomato pseudo-curly top virus* MGNLISMCLYNSKGNSTAKINDSSTWYPQP----

Q67621 TYCS2 *Tomato yellow leaf curl Sardinia virus* MGNLISTCSFSSRVNSTAKITDSSIWYPQP----

Q9QDJ8 TYLCC *Tomato yellow leaf curl China virus* MGLLTCMFSSNSKESSSVRIKDSSISHPHT----

Q96705 CALCV *Cabbage leaf curl virus* MKLFRCFKPCRGQSSNPHTSESQERNIQTG----

Q08588 ICMV *Indian cassava mosaic virus* MRMGSLICTCSSSSKANTNARISDSSTWYH----

P0C6G4 PHUV *Pepper huasteco yellow vein virus* MKMGNLICTCLFSSKENTNARTTDSLTSYP----

P0C6G5 SLCV *Squash leaf curl virus* MFPKKKFLRCFCISRGRSSNRITLESPERN----

P36283 TLCV *Tomato leaf curl virus* MRMGSLISTCLSSSKASSSARINDSSTWSP----

P0C6G2 ACMV *African cassava mosaic virus* MPFGDNYIMSPINRRRCSRVSPVECLQLTW----

CAJ7881 EACMV East African cassava mosaic virus MGCLISMFSSNSKASSNVPTpdssisfphp----

P0C6G3 PYMVV *Potato yellow mosaic virus* MGNLISTFLSSSKGNSTAQITDCSIWCPRP----

P0C2W9 TGMVY *Tomato golden mosaic virus* MGNLTSTCLFSSRENTAAKINDSSTWYPQQ----

P27271 TYLCI *Tomato yellow leaf curl virus* MGNHISMCLSNSKANTNVRTNGSSTWYPQT----

A1YKZ2 *TMHV* Tomato mosaic Havana virus MGSLISMCFYSSKPNTNARTTDSSTWAPHH----

Q7TGQ6 AYVCV *Ageratum yellow vein China virus* MGLHTCMFSSSSKENSNARIKDSSISPPQV----

AJ489258 TYLCA *Tomato yellow leaf curl virus-Almeria* MGNHISMCLSNSKANTNVRTNGSSTWYPQT----

AIN36205 BGMV *Bean golden mosaic vi*rus mgnhicmllsnskasssariaecstsntqq----
